# Benchmarking residue-resolution protein coarse-grained models for simulations of biomolecular condensates

**DOI:** 10.1371/journal.pcbi.1012737

**Published:** 2025-01-13

**Authors:** Alejandro Feito, Ignacio Sanchez-Burgos, Ignacio Tejero, Eduardo Sanz, Antonio Rey, Rosana Collepardo-Guevara, Andrés R. Tejedor, Jorge R. Espinosa

**Affiliations:** 1 Department of Physical-Chemistry, Complutense University of Madrid, Madrid, Spain; 2 Maxwell Centre, Cavendish Laboratory, Department of Physics, University of Cambridge, Cambridge, United Kingdom; 3 Yusuf Hamied Department of Chemistry, University of Cambridge, Cambridge, United Kingdom; 4 Department of Genetics, University of Cambridge, Cambridge, United Kingdom; Georgia Institute of Technology, UNITED STATES OF AMERICA

## Abstract

Intracellular liquid–liquid phase separation (LLPS) of proteins and nucleic acids is a fundamental mechanism by which cells compartmentalize their components and perform essential biological functions. Molecular simulations play a crucial role in providing microscopic insights into the physicochemical processes driving this phenomenon. In this study, we systematically compare six state-of-the-art sequence-dependent residue-resolution models to evaluate their performance in reproducing the phase behaviour and material properties of condensates formed by seven variants of the low-complexity domain (LCD) of the hnRNPA1 protein (A1-LCD)—a protein implicated in the pathological liquid-to-solid transition of stress granules. Specifically, we assess the HPS, HPS-cation–*π*, HPS-Urry, CALVADOS2, Mpipi, and Mpipi-Recharged models in their predictions of the condensate saturation concentration, critical solution temperature, and condensate viscosity of the A1-LCD variants. Our analyses demonstrate that, among the tested models, Mpipi, Mpipi-Recharged, and CALVADOS2 provide accurate descriptions of the critical solution temperatures and saturation concentrations for the multiple A1-LCD variants tested. Regarding the prediction of material properties for condensates of A1-LCD and its variants, Mpipi-Recharged stands out as the most reliable model. Overall, this study benchmarks a range of residue-resolution coarse-grained models for the study of the thermodynamic stability and material properties of condensates and establishes a direct link between their performance and the ranking of intermolecular interactions these models consider.

## Author summary

Molecular simulations have proved to be invaluable for gaining microscopic insights of the physicochemical processes underlying the formation of membraneless organelles—e.g., biomolecular condensates—in the cell. Computational efficiency and predictive accuracy are mandatory requirements for biophysical models aiming to elucidate the phase behaviour of condensates. In this study, we evaluate the performance of several state-of-the-art residue-resolution protein models in describing the phase diagram and material properties of condensates formed by different intrinsically disordered mutants of the low-complexity domain of hnRNPA1. This protein plays a key role in the stabilization and pathological solidification of the stress granules, a type of cytoplasmic condensates whose liquid-to-solid transition has been associated to the onset of multiple neurodegenerative disorders. Our results, besides serving as a benchmark for different models to describe protein liquid-liquid phase-separation, further establish a direct relation between individual intermolecular interactions and condensate phase behaviour. We conclude that the central intermolecular interactions dictating the phase behaviour of the hnRNPA1 low-complexity domain—the key protein domain involved in phase-separation and fibrillization—are predominantly cation-*π* interactions, including arginine-tyrosine and arginine-phenylalanine contacts, as well as *π*-*π* interactions mediated by tyrosine and phenylalanine contacts.

## 1 Introduction

The formation of intracellular membraneless organelles (MLOs), known as biomolecular condensates, represents an essential mechanism enabling the spatiotemporal organisation and functional regulation of the cell material [1–3]. Biomolecular condensates contain a wide variety of biomolecules, including intrinsically disordered proteins (IDPs), multi-domain proteins, and DNA or RNA strands. These condensates are thought to form via spontaneous demixing of biomolecules by means of liquid-liquid phase separation (LLPS). Biomolecular condensates have been linked to diverse biological functions, such as cell signalling [4–6], buffering protein concentrations [7, 8], compartmentalisation [7–10], genome silencing [11, 12], or noise buffering [13–15] among others [16, 17]. Many naturally occurring phase-separating proteins—such as the widely studied *fused in sarcoma* (FUS) [18–20], the TAR DNA-binding protein of 43kDa (TDP-43) [20–22], or the heterogeneous nuclear ribonucleoprotein A1 (hnRNPA1) [23, 24]—are multi-domain proteins that contain intrinsically disordered regions (IDRs). IDRs confer multi-valency to such proteins, as they can establish multiple transient intermolecular interactions, which have been consistently shown to promote condensate formation via phase separation [25–28]. Some of the IDRs present in phase-separating proteins are characterized by having amino acid sequences of low complexity, and thus are termed low-complexity domains (LCD). Besides promoting condensate formation, LCDs can also trigger the progressive rigidification of liquid-like biomolecular condensates into solid-like states [29–31]. The liquid-to-solid transition of condensates has been linked to the onset of several neurodegenerative pathologies, such as amyotrophic lateral sclerosis (ALS) [32–34], frontotemporal dementia (FTD) [35, 36], or Alzheimer [37, 38], and even to some types of cancer [17, 39, 40]. Thus, unravelling the conditions, factors, and interactions regulating protein self-assembly, and their subsequent potential liquid-to-solid transition into aberrant solid states represents an urgent challenge in cell biology [25, 34].

Uncovering the factors governing the phase behaviour of biomolecules represents a complex challenge, requiring an integrated approach that combines both experimental and computational methodologies [2, 41, 42]. Biomolecular modelling and simulations play a pivotal role in unravelling the underlying mechanisms and parameters driving to phase separation. These approaches offer detailed insights into the forces mediating interactions between biomolecules, enhancing our understanding of the processes that govern their assembly [41–43]. Biomolecular simulations can widely vary in resolution and performance of the models used, spanning from atomistic models [44–47]—where all atoms in the system are described explicitly but only a few proteins or protein segments can be studied—to coarse-grained models [48–51]—where groups of several atoms, or even whole proteins, are represented by a single particle and the interactions among particles are simplified to improve computational efficiency, allowing the investigation of systems with hundreds of biomolecules. Amongst these, sequence-dependent coarse-grained models, which have a resolution of one-bead per amino acid, have become the method of choice for probing the link between amino acid sequence and phase behaviour [41, 52–61]. These models have been used to gain insights on molecular mechanisms explaining the phase behaviour of many different proteins [62–68], as well as the impact of other biomolecules—e.g. RNA, DNA and chromatin—in controlling condensate architecture, stability, or transport properties of condensates [47, 69, 70]. Through iterative refinement and validation against experimental benchmarks, these sequence-dependent coarse-grained models are continuously advancing and provide valuable insights into the intricate relationships between protein sequence, structure, dynamics, and their collective phase behaviour [42, 52].

Experimental measurements are one of the fundamental baselines to guide the development and testing the performance of sequence-dependent models for studying protein phase behaviour [52, 53]. Approaches for the parametrization of sequence-dependent coarse-grained models include using experimentally-derived hydrophobicity scales [43, 54] machine-learning algorithms [41, 55, 57], and combining bioinformatic analyses with atomistic simulations [53, 61]. A common approach for testing the performance of sequence-dependent coarse-grained models has been comparison against *in vitro* measurements of single-molecule radius of gyration (*R*_*g*_) [71–75] of IDPs [41, 56, 59, 76]. Sequence-dependent coarse-grained models, like Mpipi [53] and Mpipi-Recharged [61], have been tested instead by comparing their predictions against experimental phase diagrams of protein solutions [77]. Therefore, experimental efforts providing temperature-dependent coexistence lines, single-protein radius of gyration, or viscosity values are enormously valuable, aside of their own interest, to assess the predictive capability of coarse-grained (CG) models [77, 78].

In this work, we benchmark the performance of six residue-resolution CG models by evaluating their predictions of the saturation concentration and temperature-vs-density phase diagrams for several hnRNPA1-LCD (referred to as A1-LCD hereafter) mutants against *in vitro* experimental data [77, 78]. The models under evaluation are: HPS [43], HPS-cation–*π* [56], HPS-Urry [54], CALVADOS2 [55], Mpipi [79], and Mpipi-Recharged [61]. Our analysis highlights the sensitivity of these coarse-grained models in capturing the effects of sequence modifications on the propensity of A1-LCD condensates to form. In addition, we test the ability of the models to capture condensate viscosity. This is a particularly challenging benchmark, as condensate viscosity is not an explicit target property in the parametrization of any of these models. Nevertheless, we consider this as a critical test since accurately capturing viscosity reflects the models’ capacity to balance the diverse biomolecular forces that stabilize condensates. Thus, how well the models predict condensate viscosity is directly linked to the quality of their parametrizations of amino acid pairwise interactions. After establishing the differences in the model predictions for the experimental phase behaviour of A1-LCD [77, 78], we analyse the protein intermolecular contact maps and the most frequent residue–residue interactions within condensates for the studied mutants, with the aim to elucidate key differences among the various models. Overall, our findings contribute to the refinement of residue-resolution coarse-grained models and describe how changes in model parametrizations impact differently a range of biophysical properties of condensates.

## 2 Results

### 2.1 Prediction of A1-LCD coexistence lines using different sequence-dependent models

The determination of the phase diagram establishes the thermodynamic conditions to investigate the formation and stability of biomolecular condensates [80, 81]. Numerous experiments have consistently shown how aromatic residues represent key ‘stickers’ to sustain protein phase separation of prion-like domain proteins [23, 77, 78, 82, 83]. Therefore, to evaluate the performance of different models such as the HPS [52], HPS-cation–*π* [56], HPS-Urry [54], CALVADOS2 [55], Mpipi [53] and Mpipi-Recharged [61] in reproducing condensate behaviour (see Section 4.1 for technical details about the models), we consider the different mutants of A1-LCD reported in Ref. [78] that display multiple variations in the type of aromatic residues along their sequence (see S1 Table to visualize the studied sequences). These variants are the following: (1) WT+NLS which corresponds to the A1-LCD wild-type sequence, which features a nuclear localization signal [78] and contains 8 tyrosines (Y) and 12 phenylalanines (F); (2) allF with 19F; (3) allY with 19Y; (4) allW with 19 tryptophans (W); (5) YtoW with 7W and 12F; (6) FtoW with 7Y and 12W; and (7) W^−^ with 13W. Importantly, the mutations in all variants are performed on the aromatic residues conserving the sequence patterning and just changing the chemical identity of the residues [78].

**Fig 1:**
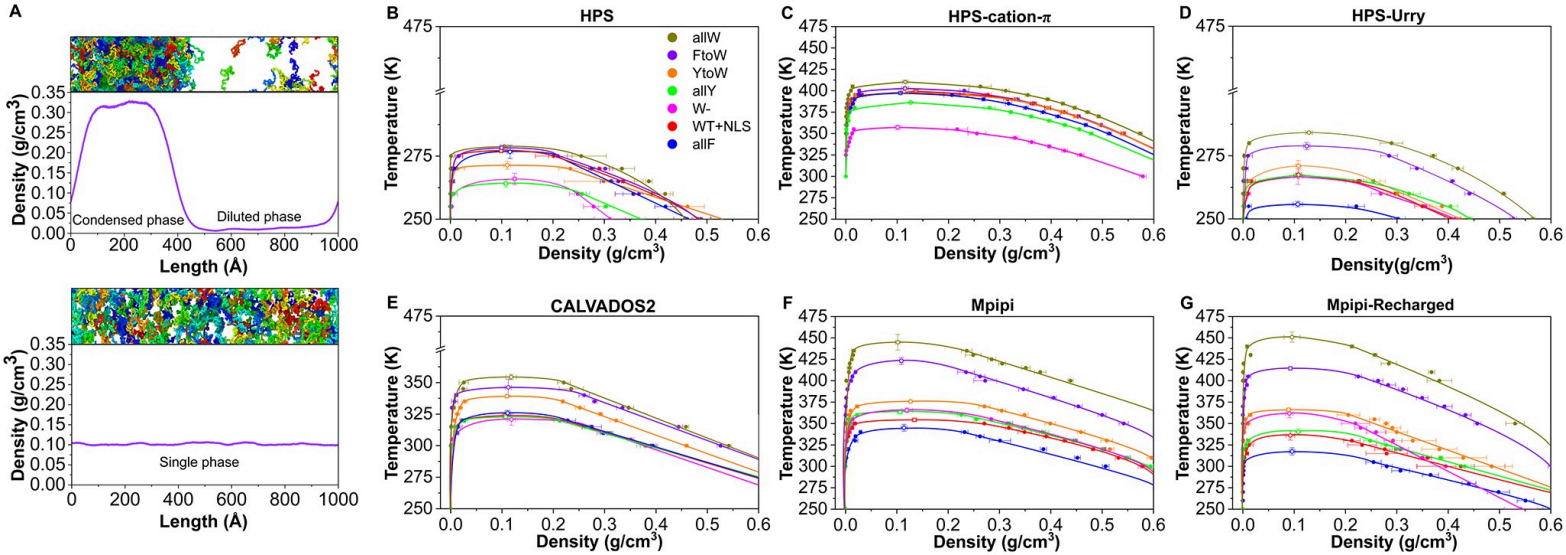
(**A**) Snapshots of a Direct Coexistence (DC) simulation used to calculate the phase diagram and critical temperature of A1-LCD (WT+NLS) protein, where each protein replica has a different colour. Top panel exhibits phase separation with a distinguishable condensed and diluted phase as depicted by the associated density profile. Bottom panel represents a system above the critical solution temperature where no phase separation occurs and a single phase is detected across the density profile. B-G Temperature–density phase diagrams of A1-LCD variants calculated via DC simulations employing the HPS (**B**), HPS-cation–*π* (**C**), HPS-Urry (**D**), CALVADOS2 (**E**), Mpipi (**F**) and Mpipi-Recharged (**G**) models. Critical temperatures are represented by empty circles while filled circles depict coexistence densities obtained from DC simulations. The lines serve as a guide to the eye. The colour coding is preserved throughout all the panels for all the variants as indicated in the legend of panel B.

We compute the temperature-vs-density phase diagrams of the aforementioned sequences by performing Direct Coexistence (DC) [84, 85] Molecular Dynamics (MD) simulations of protein solutions (see Fig 1A and Section 4.3 for further methodological details). In the DC method, the two coexisting phases are simulated by preparing periodically replicated slabs of the two phases (the condensed and the diluted phase) in the same simulation box. Once the system is equilibrated, a density profile along the long axis of the box can be extracted to compute the density (or concentration) of the two coexisting phases after a production run of ∼ 1*μ*s (please see S1 Video for a representative trajectory of our DC simulations). Importantly, we note that none of the six employed models includes explicit solvent, hence, effectively in our DC simulations the protein diluted phase corresponds to a vapour phase. In Fig 1A, a typical snapshot of the simulation slab is shown together with the associated density profile below the critical temperature (Top panel), and above the critical point (Bottom panel), where a single phase is present.

The phase diagrams for the seven A1-LCD variants, computed using six different models, are shown in Fig 1B–1G. These diagrams highlight the varying sensitivity of the models in capturing the effects of specific amino acid mutations across the protein sequence. Among the models, Mpipi and Mpipi-Recharged (Fig 1F and 1G, respectively) display the largest variation in the critical temperature (T_*c*_), with T_*c*_ spanning more than 100 K from the lowest to the highest value across the variants. In contrast, the T_*c*_ values predicted by the HPS and HPS-family models vary by only about 30 K between the lowest and highest values (Fig 1B–1D). Despite this, the HPS-Urry model (Fig 1D) demonstrates greater sensitivity to amino acid mutations, as evidenced by the larger spacing in T_*c*_ values among the variants.

The HPS model [76] represents amino acid pair interactions using a Ashbaugh-Hatch potential parametrized based on the Kapcha and Rossky (KR) hydrophobicity scale [86]. Specifically, the hydrophobicity values from the KR scale (λ_*i*_, where *i* is the amino acid) are used to define the interaction strength parameter of the Lennard-Jones term in the Ashbaugh-Hatch potential for each amino acid type. To determine the Lennard-Jones parameters for interactions between different amino acid types, the model uses the Lorentz-Berthelot mixing rules. The HPS model also incorporates salt-screened charge–charge interactions modelled through a Debye-Hückel potential. The HPS-cation–*π* model [56] is a reparametrization of the HPS model that enhances the relative strength of all cation–*π* pair interactions to better capture the experimentally observed phase separation propensity of DDX4 IDR variants [87]. More recently, the HPS-Urry model [54] was developed to improve the predictive accuracy of the HPS family of models. This version replaces the KR hydrophobicity scale with the Urry hydrophobicity scale [88], enabling improved predictions of the effects of R-to-K and Y-to-F mutations on the phase behaviour of protein solutions.

The CALVADOS family of models [41, 55], including the CALVADOS2 model tested here, builds on the foundation of the HPS model. However, instead of using a hydrophobicity scale to define the λ_*i*_ values, and subsequently the Lennard-Jones parameters, the CALVADOS models employ a Bayesian learning approach. In this approach, the λ_*i*_ values for all amino acids are optimized to achieve agreement with experimental single-molecule radii of gyration and paramagnetic relaxation enhancement (PRE) nuclear magnetic resonance (NMR) data across a broad range of intrinsically disordered regions (IDRs).

In contrast, the Mpipi model [53], developed by our group, takes a fundamentally different approach by abandoning the Lorentz-Berthelot mixing rules and instead defining amino acid pair interactions in a pair-specific manner. Additionally, the model uses the Wang-Frenkel potential, which offers greater flexibility and computational efficiency compared to the Lennard-Jones potential. For charge–charge interactions, Mpipi employs a Debye-Hückel potential, similar to the HPS and CALVADOS families. However, we recently introduced the Mpipi-Recharged model [61], which enhances the original Mpipi model by refining its description of electrostatic interactions. Specifically, Mpipi-Recharged replaces the pair-agnostic Debye-Hückel potential with a pair-specific, non-symmetric Yukawa potential. This parametrization is based on atomistic simulations of amino acid pairs in explicit water with ions, allowing for a more accurate depiction of charge effects. By abandoning mixing rules in the Wang-Frenkel potential (in both Mpipi and Mpipi-Recharged models) and incorporating amino acid-specific descriptions of charge interactions (in the Mpipi-Recharged), the Mpipi family of models addresses critical limitations of standard mixing rules. These rules may fail to capture nuanced biomolecular interactions, such as the subtle balance between aromatic stacking, cation–*π* interactions, and charge–charge interactions. Treating pair interactions explicitly enables the Mpipi models to achieve excellent agreement with experimental data [53, 61, 68, 89]. This pair-specific approach does not only improve accuracy but also provides a more detailed understanding of the molecular forces driving phase behaviour [61].

To provide additional context on the differences between the models and their resulting phase diagrams, S1 Fig presents the relative interaction strength maps for each model, encompassing both hydrophobic and electrostatic interactions between all amino acid pairs. These interaction maps reveal significant differences in how interactions are parametrized in the models for the aromatic residues Y, F, and W. In the HPS and HPS-cation–*π* models, the strength of interactions between aromatic residues and the rest of the amino acids are relatively homogeneous. In contrast, Mpipi, Mpipi-Recharged, CALVADOS2, and HPS-Urry display more pronounced differences in their energy scales, with W > Y > F in terms of interaction strength. The variations in the energy scale of the models and the relative strength of interactions among amino acid pairs strongly influence how sensitive each model is to specific (aromatic) sequence mutations and their impact on the encoded phase behaviour. Models with greater differences in interaction strengths for aromatic residues tend to show enhanced sensitivity to mutations, as reflected in their broader T_*c*_ ranges and the corresponding phase diagrams shown in Fig 1B–1G.

**Fig 2:**
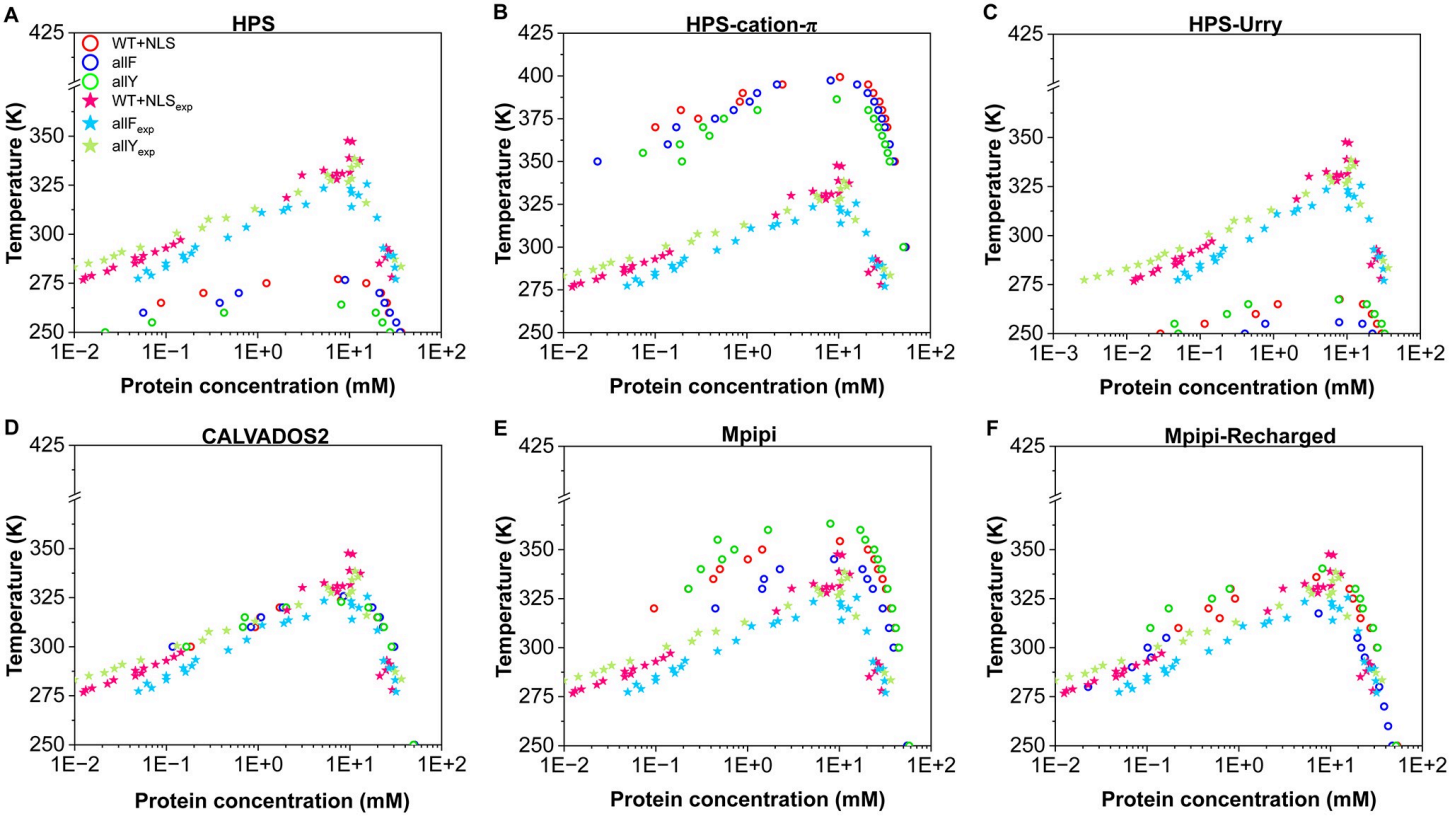
Experimental (solid stars) *vs.* simulated (empty circles) phase diagrams for the WT+NLS, allF, and allY variants of A1-LCD using different models: HPS (A), HPS-cation–*π* (B), HPS-Urry (C), CALVADOS2 (D), Mpipi (E), and Mpipi-Recharged (F).

The temperature-vs-concentration phase diagram of three of these sequences (WT+NLS, allY, and allF) has been recently measured *in vitro* [77, 78]. By fitting critical solution temperatures from the experimental data, we now establish a direct comparison between modelling and experiments so we can assess the ability of the models to predict A1-LCD phase behaviour. Accordingly, the phase diagrams in Fig 2 are plotted as a function of the protein concentration (and in logarithmic scale) to evaluate their predictive capability against experiments in the diluted phase. Determining the protein concentration in the diluted phase—specifically, the saturation concentration—poses significant challenges for Direct Coexistence simulations, particularly at low temperatures, due to the limited sampling inherent to these simulations [68].

**Fig 3:**
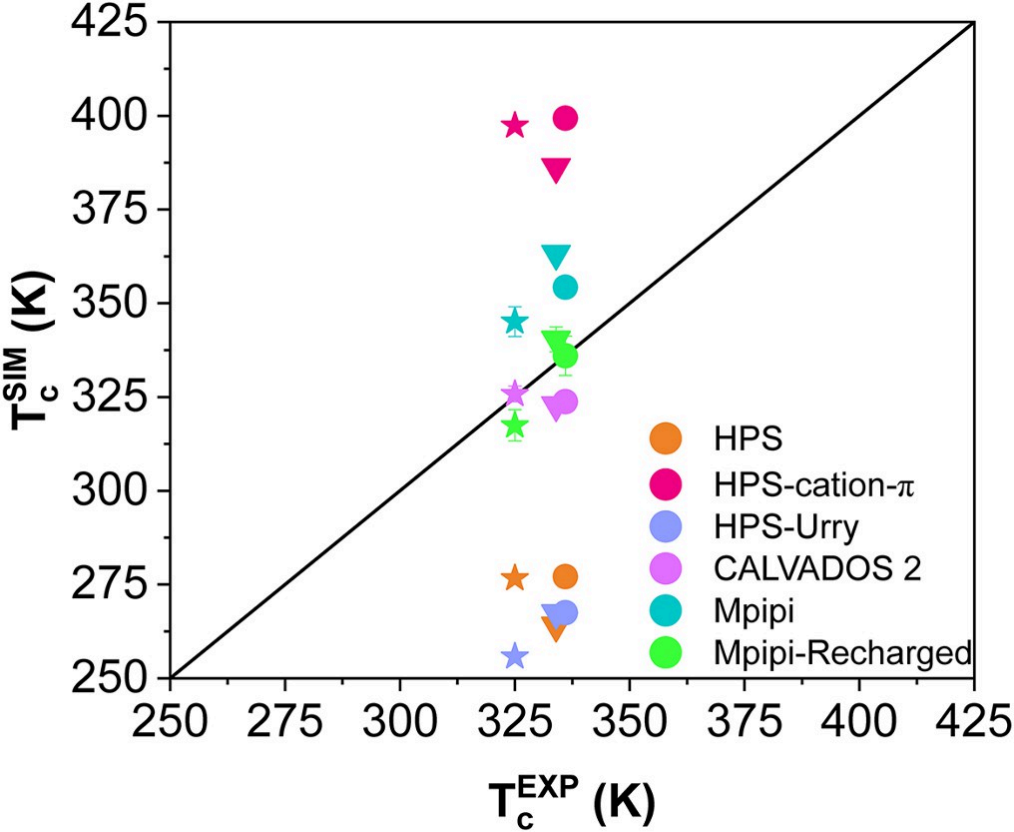
Comparison of the critical temperatures of WT+NLS (circles), allF (stars), and allY (triangles) calculated in simulations and estimated from experiments (by applying the law of rectilinear diameters and critical exponents [90] to measurements of saturation concentrations at several temperatures [77, 78]). The black solid line indicates perfect correlation between experimental and simulation results.

As illustrated in Fig 2A, the HPS model [76] consistently underestimates the critical solution temperatures for the three A1-LCD variants tested. When comparing the predicted critical temperatures from simulations (orange symbols in Fig 3) with the corresponding experimental values (derived by us from coexistence concentrations reported in Ref. [77]), the trend line deviates significantly from the perfect-fit reference (black line with a slope of 1 and intercept of 0; Fig 3). This suggests that the HPS model does not effectively capture the impact of aromatic amino acid mutations on the critical solution temperatures of the A1-LCD system. The discrepancy is likely due to the parametrization of this early version of the HPS model, which relies on the KR hydrophobicity scale [76, 86]. In the KR scale, F is ranked as more hydrophobic than W, and W is ranked as more hydrophobic than Y, potentially misrepresenting the relative contributions that these aromatic residues have in the phase separation of these systems.

In contrast, the Urry hydrophobicity scale [88] ranks the hydrophobicity of aromatic residues as W > Y > F. Consistently with this, the HPS-Urry model qualitatively captures the experimental trend in the relative phase separation propensities of the WT+NLS, allF, and allY variants, as indicated by the violet symbols in Fig 3. While the predicted order of phase separation propensities is correct, the absolute values of the critical solution temperatures predicted by the HPS-Urry model deviate significantly (by about ∼20% on average) remaining consistently lower than the fitted experimental data (Fig 2C).

The HPS-cation–*π* model [56] predicts higher-than-expected critical temperatures (approximately ∼30% higher on average) for the three variants (Figs 2 and 3). This discrepancy can be attributed to a substantial overestimation of cation–*π* interactions in the HPS-cation–*π* model (S1 Fig). Importantly, the high discrepancy in the predicted critical temperature values between the HPS-cation–*π* and HPS/HPS-Urry models highlights the prominence of cation–*π* interactions within A1-LCD condensates.

The CALVADOS2 model predicts coexistence lines that are, on average, very close to the experimental values (critical solution temperatures within approximately ∼5% of the values fitted by us from experiments). While the values are very close to the experimental ones, the relative ordering among the three variants deviates from the experimental trend (pink data in Fig 3). It is important to note that the CALVADOS2 model was not specifically designed to predict critical solution temperatures, and this evaluation goes beyond its intended scope. In this context, a recent study by our group [89], consistent with prior research [41, 55, 91], emphasizes that models developed to capture single-molecule properties of IDRs do not necessarily reproduce condensate coexistence lines. While diverse parametrizations can yield reasonable predictions for radii of gyration for diverse IDRs, the accurate prediction of phase diagrams represents a far more rigorous and demanding validation test [89].

The Mpipi model has demonstrated excellent performance in describing the phase behaviour of prion-like domain proteins [53, 68]. Consistently, our tests show that Mpipi accurately predicts the order of the critical solution temperatures for the WT+NLS, allF, and allY variants (cyan symbols in Fig 3). This agreement arises from the Mpipi model’s parametrization, which ranks the interactions of the three aromatic residues as W > Y > F. Despite its qualitative accuracy, Fig 2E reveals that Mpipi slightly overestimates the critical solution temperatures for all three variants, with an average deviation of approximately 6%. Importantly, our analysis reveals that the improved Mpipi-Recharged model achieves the highest level of accuracy in reproducing experimental values, successfully capturing both the trend and the absolute critical solution temperatures for the three variants with deviations of less than ∼3% (green symbols in Fig 3).

Overall, this analysis demonstrates that CALVADOS2, Mpipi, and Mpipi-Recharged offer a robust and accurate description of the condensate phase behaviour for A1-LCD aromatic mutants. In contrast, while the HPS-Urry model qualitatively captures the experimental phase-separation tendencies, it exhibits significant deviations in its quantitative predictions of critical solution temperatures.

### 2.2 Comparison of the phase-separation saturation concentration between experiments and simulations

The concentration threshold above which biomolecular phase separation becomes thermodynamically favourable at a given temperature—C_*sat*_(T)—is one of the critical quantities that can be controlled by cells to trigger condensate formation and dissolution on demand [7, 92]. In that sense, *in vitro* studies are extremely useful to characterise such threshold, determining the border between a single homogeneous phase and the emergence of phase separated condensates [77, 93–95]. Most of these studies have focused on measuring C_*sat*_ at physiological conditions of salt, pH, and temperature [22, 24, 93, 95–100]. However, recent studies have also reported how C_*sat*_ varies with temperature and measured the highest temperature values beyond which phase separation is no longer achievable [77, 78]. The saturation concentration quantifies the ability of a given protein to phase separate, thus, the higher the value, the lower propensity to form condensates [22, 24, 93, 97]. Similarly, for condensates that present upper critical solution temperatures (herein critical temperature), the critical temperature evaluated through DC simulations serves as a direct indicator of the ability of a protein to undergo phase separation [69]. Indeed, the *in vitro* experimental values of saturation concentrations of the A1-LCD variants [77] seem to correlate inversely with the critical solution temperatures that we can fit from the experimental coexistence densities [77]. A higher critical temperature is directly indicative of greater thermodynamic stability of the condensates. Thus, we can correlate the critical temperature obtained from simulations to the experimental saturation concentration at a given constant temperature (e.g., 298 K) to obtain a qualitative overview of the models sensitivity for describing protein phase behaviour as a function of specific sequence mutations [61, 69].

**Fig 4:**
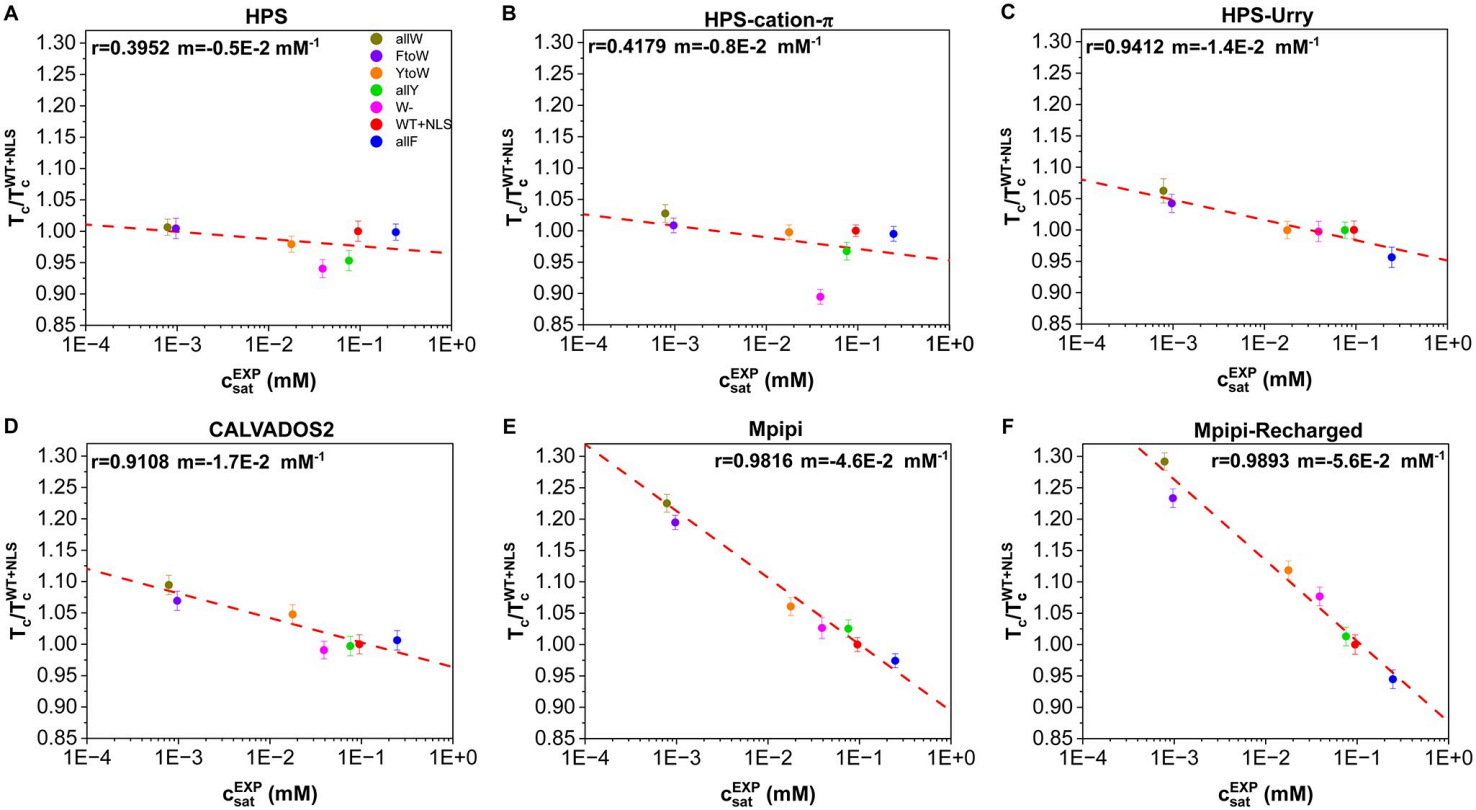
Predicted critical temperature (normalised by the critical temperature of the WT+NLS sequence) for the different models HPS (A), HPS-cation–*π* (B), HPS-Urry (C), CALVADOS2 (D), Mpipi (E), and Mpipi-Recharged (F) *vs.* the experimental saturation concentration at 298 K reported in Ref. [78] for the different A1-LCD mutants. The panels include the Pearson correlation coefficient (*r*) and the slope (*m*) from a linear fit to the data. The error bars show the uncertainty in the critical temperature associated to its calculation using the laws of critical exponents and rectilinear diameters (see further details in Section 4.3).

In Fig 4, we show the simulated critical temperatures (normalised by the critical temperature of WT+NLS) against the experimental saturation concentrations [78] measured at 298 K for the different A1-LCD mutants. The experimental C_*sat*_ vary significantly, changing by more than 2 orders of magnitude among the variants studied [78]. Thus, the comparison we perform in this section tests the sensitivity of the six different models to describe the impact of mutations on modulating the thermodynamic stability of A1-LCD condensates (Fig 4A–4F).

To compare the predictions of the various models, we establish a linear relationship between the normalised critical solution temperatures and the experimental saturation concentrations, expressed as $T_{c}/T_{c}^{WT+NLS}=mC_{sat}^{EXP}+b$, where *m* is the slope and *b* is a constant. We use *r* (the Pearson correlation coefficient) to quantify the quality of the fit. Notably, the HPS and HPS-cation–*π* exhibit the smallest absolute values for the slope *m* in this relationship, ranging from −0.5 × 10^−2^ to −0.8 × 10^−2^mM^−1^. This indicates that the critical temperatures predicted by these two models are only weakly affected by the mutations studied here (Fig 4A and 4B, respectively). Furthermore, the quality of the fit is relatively low for the HPS (*r* = 0.3952) and HPS-cation–*π* models (*r* = 0.4179), suggesting a poor correlation between their predicted critical temperatures and the experimental trends for C_*sat*_.

The Mpipi and Mpipi-Recharged models predict the most significant changes in critical temperature as a function of the variations in saturation concentration among the variants (Fig 4E and 4F, respectively). This is reflected in the steep values of their slopes, −4.6 × 10^−2^ and −5.6 × 10^−2^mM^−1^, respectively. Additionally, both models exhibit the strongest linear correlation with the experimental data, achieving Pearson correlation coefficients exceeding 0.98.

Finally, the HPS-Urry and CALVADOS2 models exhibit intermediate performance compared to the HPS/HPS-cation–*π* and Mpipi/Mpipi-Recharged models. The critical temperatures predicted by both HPS-Urry and CALVADOS2 display a strong linear correlation with the experimental C_*sat*_ values, achieving Pearson correlation coefficients above 0.91. However, both models predict only a moderate decrease in *T*_*c*_ as a function of changes in the saturation concentration of the variants studied (Fig 4C and 4D). Because the CALVADOS2 model was not specifically designed to predict critical solution temperatures, this evaluation extends beyond the model’s intended scope.

Building on these results, we now focus on the CALVADOS2, Mpipi, and Mpipi-Recharged models to further investigate the phase behaviour of A1-LCD mutants. Specifically, we use these models to compute the saturation concentration at 298 K (C_*sat*_) by extensively sampling the equilibrium protein concentration in the diluted phase in coexistence with the condensates through Direct Coexistence simulations. Estimating C_*sat*_ using this approach is computationally demanding due to the inherent challenges of sampling the diluted phase, particularly at low temperatures [68]. The diluted phase contains a very low concentration of molecules, meaning there are intrinsically few particles present. This low number of particles can result in significant variability in the measured concentrations across different regions of the simulation box, introducing substantial noise into the measurement. Furthermore, exchanges of molecules between the diluted and condensed phases are rare events, which require long simulation timescales to be adequately captured. These challenges are exacerbated at lower temperatures, where the equilibrium concentration in the diluted phase becomes even smaller. While computationally demanding, extracting C_*sat*_ from the DC method allows us to perform a direct comparison between the same observable measured in both simulations and experiments.

**Fig 5:**
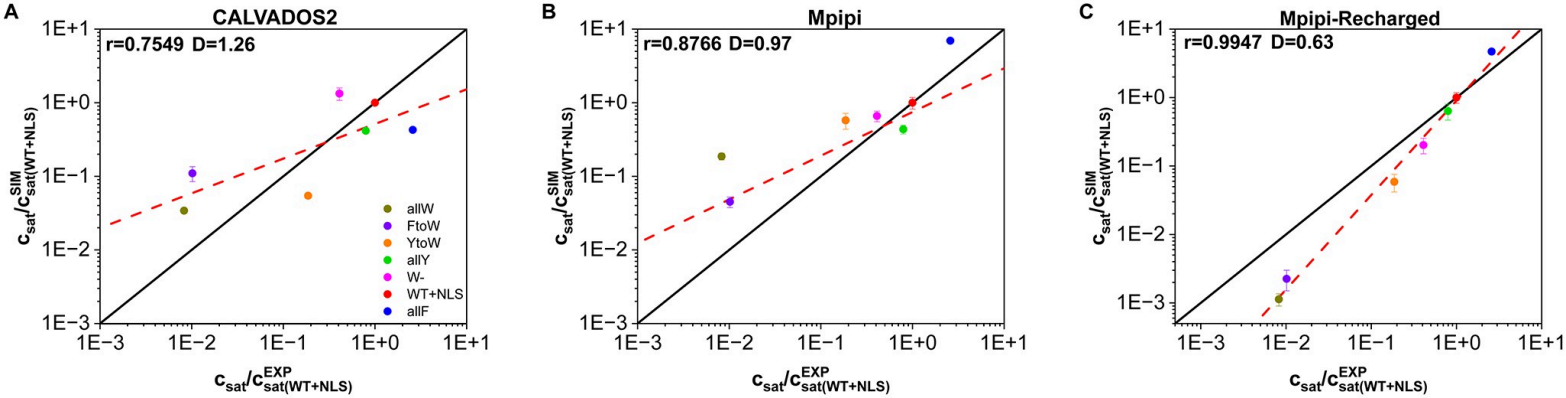
Simulated *vs.* experimental saturation concentration (C_*sat*_), normalised by the WT+NLS C_*sat*_ [78], for the different variants and for the models CALVADOS2 (A), Mpipi (B) and Mpipi-Recharged (C). The Pearson correlation coefficient (*r*) and the root mean square deviation from the experimental values (*D*) are displayed for each set of simulation data. The black lines indicate a perfect match between experimental and computational values, while the red dashed lines depict a linear regression for each set of data.

In Fig 5, we present the predicted saturation concentration values from simulations, normalised by the saturation concentration of the WT+NLS sequence, plotted on a logarithmic scale. The predictions from the CALVADOS2, Mpipi, and Mpipi-Recharged models are compared against experimentally determined normalised saturation concentrations for the studied mutants (($C_{sat}^{EXP})/\left(C_{sat(WT+NLS}^{EXP}\right)$; see Section 4.6 for further information) reported in Ref. [78]. To quantify the agreement between the simulations and experiments, we calculate *D*, a metric that measures the average deviation of the simulation predictions from the experimental values (details provided in Section 4.6). Lower *D* values indicate closer agreement with the experimental data. Additionally, we perform a linear fit to examine the relationship between the normalised simulation results and the experimental data (red dashed line in Fig 5), comparing it to the perfect fit (diagonal black line in Fig 5). To assess the strength of the linear correlation, we calculate the Pearson correlation coefficient (*r*). This metric evaluates whether the models qualitatively capture the experimental trend, even if quantitative deviations are present.

We find that the prediction of the Mpipi-Recharged model for the normalised C_*sat*_ presents the lowest average deviation, *D* = 0.63, with respect to the experimental values and the highest Pearson correlation coefficient among the set (*r* = 0.9947), indicating exceptional agreement with the experimental trends. The Mpipi and CALVADOS2 models also provide good descriptions with mean deviations of *D* = 0.97 and *D* = 1.26, respectively, and Pearson correlation coefficients of *r* = 0.8766 and *r* = 0.7549, respectively.

In addition, we compare the absolute experimental and simulation *C*_*sat*_ values (S2 Fig). In this comparison, the values of the Pearson correlation coefficients remain unchanged from those in Fig 5. That is, the predictions of the Mpipi-Recharged model exhibit the strongest correlation with the experimental values of *C*_*sat*_, followed by Mpipi, and finally CALVADOS2. In contrast, the size of the mean errors, *D*, do change, revealing that CALVADOS2 provides *C*_*sat*_ with the smallest overall error (*D* = 0.38), followed by the Mpipi-Recharged (*D* = 0.78) and finally the Mpipi model (*D* = 0.93).

Overall, our comprehensive evaluation in this Section shows that all three models—CALVADOS2, Mpipi, and Mpipi-Recharged—perform exceptionally well in predicting the phase-separation propensity of A1-LCD variants, with Mpipi-Recharged standing out as the most accurate potential. Their excellent performance is particularly noteworthy given the substantial challenges coarse-grained models face in reproducing experimental coexistence lines.

### 2.3 Condensate viscoelastic behaviour of A1-LCD mutants by different models

Condensate viscoelastic properties have been linked to the roles of these systems in health and disease [34, 101, 102]. Whereas liquid-like states in condensates are associated to biological function, their progressive transition into solid and gel-like states has been linked to the onset of multiple neurological disorders [24]. Examples of protein condensates displaying hardening over time have been reported for RNA-binding proteins such as FUS [103, 104], TDP-43 [105], or hnRNPA1 [106] among others [30]. Hence, it is important to determine the ability of different residue-resolution CG models to probe the material properties of condensates: i.e., whether they behave as liquids or gels, and whether their viscosity remains constant or increases over time [31]. Moreover, determining the ability of residue-resolution CG models to characterise which precise interactions and domains control their time-dependent transport properties [103, 107–109] is central. Such information is essential to understand the molecular onset between functional and dysfunctional behaviour.

Computationally, the generalised Green-Kubo relation [110, 111] (see Section 4.5) is one of the most efficient methods for evaluating viscosity of protein condensates using residue-resolution CG models [31, 69, 112]. Calculating the shear stress relaxation function (*G*(*t*)) using the Green-Kubo approach provides information of both inter- and intra-molecular protein interactions, which may vary significantly with the chosen CG model [69, 108, 112]. Since the predictive accuracy of most residue-resolution CG models has been optimized and tested using experimental single-protein radius of gyration and phase diagrams (either saturation concentrations or critical solution temperatures) [41, 43, 53, 54, 59], evaluating their ability to predict condensate viscosity provides a demanding benchmark that extends beyond the scope of their original parametrizations.

As the experimental reference, we use the *in vitro* viscosity measurements at *T* ∼ 298 K from Ref. [78] for the seven variants of A1-LCD studied in the previous sections. We only test the performance of the CALVADOS2, Mpipi, and Mpipi-Recharged models because 1) HPS and HPS-cation–*π* failed to recapitulate the relative phase-separation propensity of the A1-LCD variants (Fig 4A and 4B, respectively); and 2) HPS and HPS-Urry do not phase-separate at 298 K, where condensates experimental viscosities have been reported.

**Fig 6:**
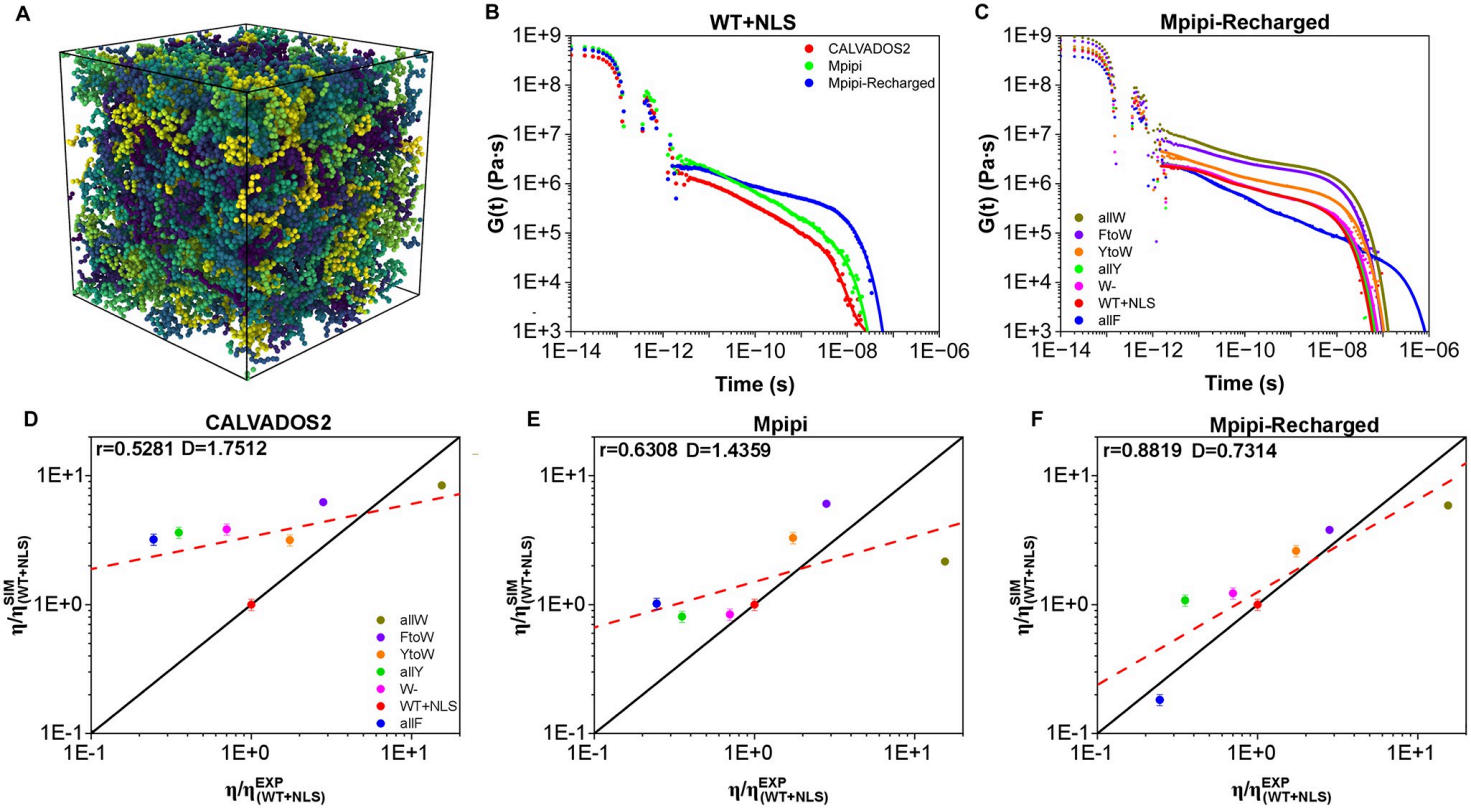
(**A**) Snapshot of a bulk NVT simulation with 200 A1-LCD (WT+NLS) protein replicas (each of them depicted by a different tone of colour) employed to compute the condensate viscosity. (**B**) Shear stress relaxation modulus (*G*(*t*)) for the WT+NLS sequence at 298 K evaluated by different models. (**C**) *G*(*t*) for different A1-LCD mutants using the Mpipi-Recharged model. In both **B** and **C** panels, *G*(*t*) raw data obtained during the simulations are represented by filled symbols, while Maxwell’s mode fits to the data (as described in Section 4.5) are plotted with solid lines. **D**, **E**, and **F**: Predicted versus *in vitro* viscosity (both reduced by the corresponding WT+NLS value) for all variants under study and the CALVADOS, Mpipi, and Mpipi-Recharged models respectively. The meaning of *r*, *D*, and the red dashed and black solid lines is the same as in Fig 5.

To estimate the viscosity, we calculate the shear stress relaxation function, *G*(*t*), of the different A1-LCD variants under bulk conditions at 298 K (see Fig 6A and S2 Video for representative configurations of such simulations, and Section 4.2 for the simulation details). For these calculations, we perform bulk simulations (in the canonical ensemble) of ∼ 3 − 5*μ*s depending on each specific system as reported in Section 4.2.

The calculation of *G*(*t*) for the WT+NLS sequence using the three models is shown in Fig 6B. While the predictions of the three models for *G*(*t*) at short timescales are rather similar, indicating that bond, angle, and intramolecular conformational relaxation is similar for all of them, we observe large differences at long times. At long timescales, the slope and terminal decay of *G*(*t*), which strongly determines the condensate viscosity, differs significantly among the three models, clearly demonstrating the critical influence of the model parametrization on the resulting viscoelastic properties of the condensates. Such variability is even more pronounced when comparing the behaviour of *G*(*t*) predicted by the three models for all the different A1-LCD mutants. We show the full *G*(*t*) plots predicted by the Mpipi-Recharged model in Fig 6C, and the viscosity values for the three models in Fig 6D–6F. The calculation of *η* from *G*(*t*) consists in integrating the shear stress relaxation modulus (*G*) as a function of time (for further details on this calculation see Section 4.5).

Fig 6C reveals that the amino acid mutations tested lead to drastic variations in the condensate viscosity predicted by the Mpipi-Recharged (of approximately two orders of magnitude), in perfect agreement with the experimental observations [78]. This results highlights the strong influence that specific sequence mutations have on regulating the transport properties of condensates, as previously found for FUS [113], TDP-43 [105], and Tau [114, 115].

We next compare the viscosity (*η*) values for all A1-LCD sequences as predicted by simulations using the CALVADOS2 (Fig 6D), Mpipi (Fig 6E), and Mpipi-Recharged (Fig 6F) models against the corresponding experimental values [78]. Both simulation and experimental viscosity values are normalised by the WT+NLS *η* value to enable a relative comparison. This normalisation is necessary because the absolute values predicted by all the residue-resolution coarse-grained models systematically underestimate the experimental viscosities by several orders of magnitude [69]. This discrepancy arises naturally from the implicit treatment of solvents and ions, as well as the neglect of atomic vibrations and detailed inter-atomic interactions among amino acids, due to the amino acids being represented as spherical beads. While the choice of the A1-LCD WT+NLS variant used to normalise the viscosities influences the absolute deviation between simulations and experiments, it does not alter the overall trend.

We observe that the sensitivity exhibited by the different models in their predictions of critical temperatures (Fig 4D–4F) is similarly reflected in the variation of condensate viscosity as a function of sequence mutations (Fig 6D–6F). The Mpipi-Recharged model exhibits the greatest sensitivity, with viscosity values spanning nearly two orders of magnitude among the different A1-LCD variants, and emerges as the best fit for capturing the viscoelastic behaviour of A1-LCD condensates. Specifically, the Pearson coefficient (*r*) assessing the strength of the correlation between simulation and experimental *η* values is high for Mpipi-Recharged (*r* = 0.8819), and moderate for Mpipi(*r* = 0.6308) and CALVADOS2 (*r* = 0.5281). In addition, the root mean squared deviation (*D*) describing the mean error from the experimental data is the smallest for the Mpipi-Recharged (*D* = 0.7314), and moderate for both Mpipi (*D* = 1.4359) and CALVADOS2 (*D* = 1.7512) models.

**Fig 7:**
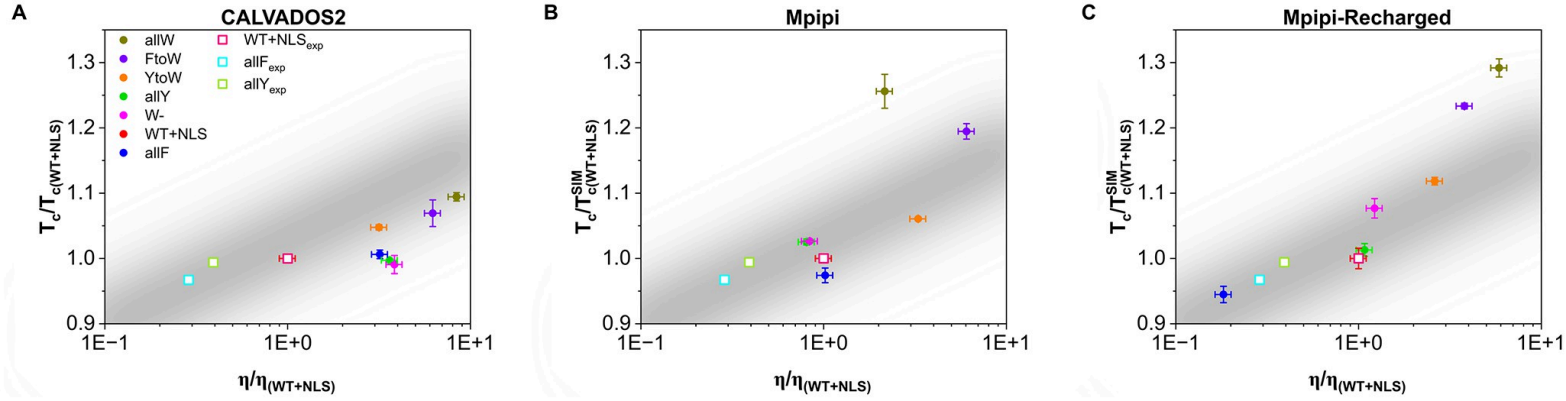
Correlation between the critical temperature (normalised by the T_*c*_ of the WT+NLS sequence) and the condensate viscosity (also normalised by *η* of the WT+NLS) for the different studied A1-LCD mutants as predicted by the CALVADOS2 (A), Mpipi (B), and Mpipi-Recharged (C) models. Experimental data from Ref. [78] for the WT+NLS, allF and allY sequences have been also included as empty squares (see legend). A linear trend to the experimental correlation between these two quantities for the three sequences measured *in vitro* [78] is shown with a grey band.

We also investigate whether a correlation between the critical temperature and condensate viscosity can be established for the different studied variants. A correlation between these two quantities is expected since intermolecular interaction strength, which favours condensation (i.e., increases T_*c*_), should also diminish molecular mobility (i.e., increasing *η*). In Fig 7, we plot the critical temperature (normalised by the T_*c*_ of the WT+NLS sequence) *vs.* condensate viscosity (also normalised by the *η* of the WT+NLS sequence) for the different studied A1-LCD mutants as predicted by CALVADOS2 (A), Mpipi (B), and Mpipi-Recharged (C) models. Moreover, experimental *in vitro* data from Ref. [78] for the WT+NLS, allF and allY sequences are included (empty squares). As can be seen, the Mpipi-Recharged predicts the correlation experimentally found for these three variants (depicted by a grey band linearly extrapolated). The full phase diagram (including T_*c*_) for the rest of the variants was not reported in Ref. [78], nevertheless, it is expected that a similar correlation as that predicted by the Mpipi-Recharged model holds since it reasonably predicts the experimental critical temperature, saturation concentration, and condensate viscosity of all these variants, as shown in Figs 2, 5 and 6, respectively. The CALVADOS2 also shows a correlation between the critical temperature and viscosity (Fig 7A). However, the CALVADOS2 predictions do not span the same range of values for viscosity as the experimental measurements (Fig 6D). For the case of Mpipi, a weak correlation between the critical solution temperature and the viscosity is observed (Fig 7B).

Future work exploring whether a correlation between the critical solution temperature and the viscosity arises in other condensates would be highly valuable, as both properties are challenging to measure and are rarely reported under identical conditions [77, 78]. Establishing such a relationship could enable the qualitative inference of one property from the other, offering complementary insights into the stability and transport properties of biomolecular condensates.

### 2.4 Key intermolecular interactions in A1-LCD condensates

Computational approaches can contribute to a deeper understanding of biomolecular phase separation by evaluating the underlying molecular information that is hardly accessible in experiments [69, 74, 116]. A powerful example is the calculation of intra and inter-molecular contact maps [52, 69, 89] which provide microscopic insights about the key amino acids and protein domains enabling biomolecular self-assembly. However, the outcome of this type of analysis is intimately related to the specific model characteristics and its interaction parameters. Therefore, different models may provide notably different contact domain probabilities for the same protein condensate.

Analyzing the differences in the inter-molecular contact maps predicted by the different models we benchmark here, also allows us to understand the reasons why such models present different performance in these benchmarks. Specifically, we compute the number of times a pair of amino acids belonging to different proteins within the condensate are in contact, where ‘in contact’ is defined as being closer than a cut-off distance which depends on the identity of the amino acids in the pair (see Section 4.4 for technical details on this calculation). We then evaluate the energy contribution of each contact considering the sum of the potential energy terms in the model at the interacting distance. That is, the sum of the Wang-Frenkel and Yukawa potentials for the Mpipi-Recharged model, Wang-Frenkel and Debye-Hückel for Mpipi, and Lennard-Jones and Debye-Hückel for CALVADOS2 and the HPS family (see Section 4.4). The resulting contact energy maps reveal the key intermolecular interactions that sustain the liquid network within the different condensates.

When comparing the contact energy maps for WT+NLS condensates predicted by the different models at 0.95 T_*c*_ (S3 Fig), we find significant differences among the behaviour of the various models. The HPS and HPS-Urry models present homogeneous maps of intermolecular interactions, with most amino acid pairs presenting contact energies that differ by at most 0.002 kJ/mol. In contrast, the HPS-cation–*π*, Mpipi and Mpipi-Recharged models give rise to heterogeneous maps, where the contact energies among amino acid pairs can differ by more than 0.004 kJ/mol. CALVADOS2 presents an intermediate behaviour between the two extremes. Such difference can be explained by the HPS and HPS-Urry models considering electrostatic and *π*-*π* interactions as stronger contributors to biomolecular phase separation than cation–*π* contacts (see S4 Fig, and the relative interaction strength maps in S1 Fig). In the CALVADOS2 model, such a difference is present, but is much more moderate [55]. The HPS-cation–*π*, Mpipi, and Mpipi-Recharged models predict that phase separation is most strongly contributed by some specific prion-like sub-domains. Whilst all models show a strong interacting region from the 105th to 115th residue (a sub-domain enriched in Y, F, and G), the HPS-cation–*π*, Mpipi, and Mpipi-Recharged also present a highly interacting sub-domain from the 130th amino acid to the 135th residue, which contains 2 arginines and 1 phenylalanine. In addition, the HPS and HPS-cation–*π*, but more prominently the Mpipi, and Mpipi-Recharged models show two sub-domains between the 75th and 85th position (enriched in asparagine and aromatic residues) and between the 15th and 45th (rich in N, F, R and G) that significantly contribute to the stability of A1-LCD condensates through cation-*π* and *π*-*π* interactions (S3 Fig).

**Fig 8:**
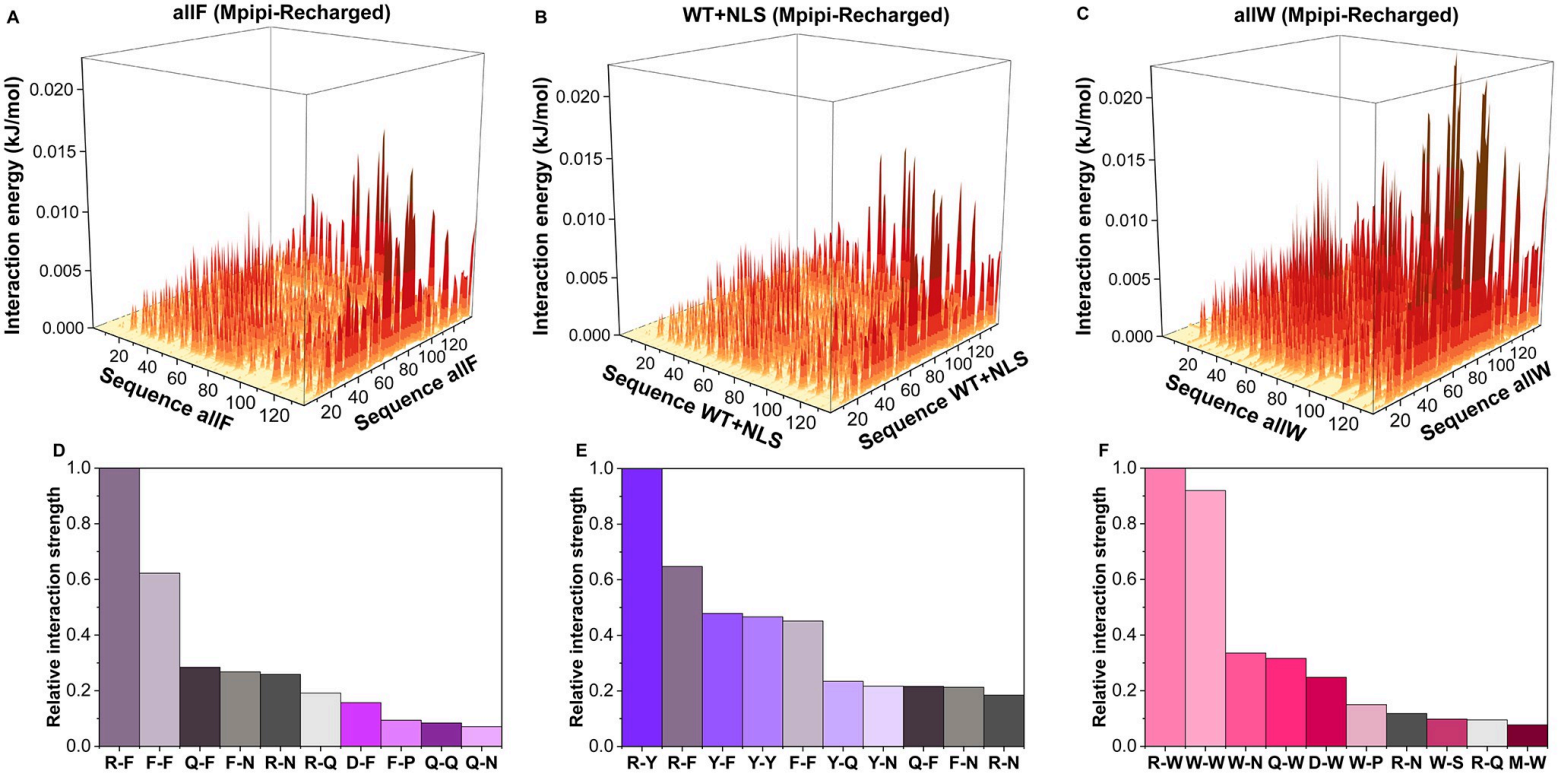
Contact energy maps of pairwise intermolecular interaction energy for allF (**A**), WT+NLS (**B**), and allW (**C**) condensates at T = 0.95 T_*c*_ as predicted by the Mpipi-Recharged model. The most frequent intermolecular amino acid pairwise interactions sustaining the condensates of allF (**D**), WT+NLS (**E**), and allW (**F**) are also displayed. Pairwise contacts have been both normalised by the highest value of residue-residue pairwise interactions and their abundance across their sequence (see Section 4.4 for further details on these calculations).

Since the Mpipi-Recharged model has consistently emerged as the most accurate model of those we have tested (Figs 1–6), we now employ it to compare the impact of amino acid mutations on the contact energy maps of A1-LCD condensates. In Fig 8, we present the contact energy maps for condensates of the WT+NLS, allF, and allW sequences at 0.95 T_*c*_, where T_*c*_ represents the critical temperature specific to each variant (Fig 8A–8C). Notably, the contact energy maps for the allF and WT+NLS condensates exhibit striking similarities (Fig 8A and 8B). In contrast, the contact energy map for the allW condensates (Fig 8C) reveals significantly stronger contact interactions among residues near the protein terminal region, particularly from the 90th residue onward. This increase in intermolecular contacts at the protein terminal region enhances the interaction energies across the whole protein sequence, suggesting a cooperative mechanism that reinforces the condensate assembly (Fig 8C). This increase of intermolecular interactions in the allW mutant explains the large increase in viscosity observed both computationally and experimentally (Fig 6) for this variant with respect to the WT+NLS and allF sequences.

We now extract from Fig 8A–8C the most frequent amino acid pairs that stabilize the condensed phase, as predicted by the Mpipi-Recharged model (Fig 8D–8F). The relative interaction strength of each pair has been normalised by the contact pair with the highest energetic contribution. As expected, the key intermolecular interactions in WT+NLS condensates are primarily cation–*π* (e.g., R–Y and R–F) and *π*–*π* interactions (e.g., F–F, Y–F, and Y–Y). In allF condensates, the dominant contacts include cation-*π* (R–F) and *π*–*π* interactions (Y–F and F–F), along with interactions involving asparagine (N) with arginine (R) and phenylalanine (F). For allW condensates, the most prevalent interactions are W–W and W–R, followed by contacts between tryptophan (W) and asparagine (N), glutamine (Q), and aspartic acid (D).

These findings align with the stickers-and-spacers framework for protein phase transitions, in which multivalent proteins are conceptualized as heteropolymers comprising ‘stickers’ (binding sites for associative interactions) and ‘spacers’ (regions between stickers) [117, 118]. Within the stickers-and-spacers framework, as proposed in Ref. [77] for A1-LCD condensates, tyrosine (Y), phenylalanine (F), and tryptophan (W) function as the primary stickers, arginine (R) acts as a context-dependent sticker, and other amino acids serve as spacers. Our simulations demonstrate that the predictions of the Mpipi-Recharged model are in excellent agreement with this framework (Fig 8D–8F).

We now focus on the most frequent interactions in WT+NLS condensates, as predicted by the Mpipi-Recharged (Fig 8E) and the CALVADOS2 model (S4D Fig). While the Mpipi-Recharged suggests that cation–*π* interactions such as R–Y and R–F are stronger contributors than Y–Y, F–F, and Y–F to the stability of A1-LCD condensates, the CALVADOS2 proposes that instead *π*–*π* contacts are more energetically favourable than cation–*π* interactions for these systems. As in the Mpipi-Recharged model, according to the Mpipi model, the five most energetically favourable contacts in A1-LCD condensates are R–Y, R–F, Y–F, F–F, and Y–Y (S4E Fig). In contrast, the HPS and HPS-Urry models do not predict cation–*π* interactions as main contributors for A1-LCD phase-separation (S4A and S4C Fig). Instead, HPS and HPS-Urry consider that electrostatic R–D and K–D contacts alongside *π*–*π* interactions (such as Y-F, F-F, and Y-Y, which in the model parametrization have a considerable interaction strength [76], see S1 Fig) are the key contacts driving biomolecular phase separation. The underestimation of cation–*π* interactions for biomolecular phase transitions was addressed by the HPS-cation–*π* reparametrization [56], which added an extra potential term to the model (see Section 1 for further details). Nevertheless, as shown in S4B Fig, the HPS-cation–*π* reparametrization significantly overestimates cation–*π* contacts predicting that R–Y, R–F, K–Y and K–F contribute four times more than aromatic interactions such as F–F or Y–F. Such overestimation of cation–*π* interactions by the HPS-cation–*π* model leads to very high critical temperatures for A1-LCD condensates, as shown in Fig 2B; this has been previously discussed in Refs. [31, 53, 56, 69, 87] for other RNA-binding proteins such as FUS, TDP-43, or DDX4 among others.

Overall, this analysis reveals that residue-resolution CG models considering both cation–*π* and *π*–*π* interactions as primary contributors to the stability of A1-LCD condensates align most closely with experimental observations. Notably, models such as Mpipi and Mpipi-Recharged, which emphasize the stronger contribution of cation–*π* interactions over *π*–*π* interactions, show the highest level of agreement with experimental data.

## 3 Discussion

Computer simulations are a powerful tool to complement *in vitro* and *in vivo* investigations of biomolecular condensates [42, 119]. We systematically compare the predictions of several models against experimental coexistence lines, critical solution temperatures fitted from experimental coexistence densities, experimentally measured saturation concentrations, and experimentally measured viscosities for A1-LCD condensates, and seven mutants. Our results show that the Mpipi-Recharged model displays quantitative accuracy in predicting the critical temperature and condensate saturation concentration of the different A1-LCD mutants (Figs 2 and 5) reported in Refs. [77, 78].

Furthermore, evaluating condensate viscosity provides a stringent test of the ability of these models to predict the biophysical properties of condensates beyond stability. Our analysis reveals that the CALVADOS2, Mpipi, and especially the Mpipi-Recharged model, accurately capture the relative variations of the viscoelasticity of protein condensates as a function of specific sequence mutations (Fig 6). This highlights the potential of residue-resolution coarse-grained models to effectively predict how sequence variations influence the material properties of condensates beyond the systems tested here.

Importantly, characterizing viscoelasticity is essential for distinguishing between liquid-like and solid-like behaviour of condensates, which is closely tied to biological function and pathological malfunction [24, 34, 42, 101, 102]. Notably, we observe a clear correlation—both emerging from the experimental and computational data (Fig 7)—between condensate thermodynamic stability (i.e., T_*c*_) and viscoelasticity. This correlation suggests that higher critical temperatures for phase separation are linked to increased condensate viscosities. This relationship emerges because both greater stability and viscosity are promoted by stronger intermolecular interactions: stronger biomolecular interactions promote condensation and, simultaneously, reduce molecular diffusivity, thereby enhancing viscosity [31, 103].

Finally, we have evaluated the intermolecular contact energy maps of A1-LCD condensates as predicted by the different models studied here, paying particular attention to the Mpipi-Recharged force field (Fig 8). Our analysis explains how the variations in the predictions of the different models may be explained by the differences in the pairwise residue-residue interactions that sustain A1-LCD condensates.

The best-performing models in our benchmarks—namely CALVADOS2, Mpipi, and the Mpipi-Recharged model—consider in their parametrizations that both cation–*π* interactions (e.g., R–Y and R–F) and *π*–*π* contacts (e.g., F–F, Y–F, and Y–Y) are the dominant intermolecular forces stabilising A1-LCD condensates. Among these models, Mpipi-Recharged, followed by Mpipi, stand out as the most accurate, particularly because they rank cation–*π* interactions as stronger contributors than *π*–*π* contacts. In contrast, models that underestimate the contribution of cation–*π* interactions—such as the HPS model—perform less accurately, failing to capture the significant shifts in critical temperatures and saturation concentrations caused by aromatic residue mutations (S4 Fig). While the HPS-cation–*π* model overhauls the contribution of cation–*π* interactions, it overemphasizes their strength, resulting in predictions of unrealistically high critical temperatures (Fig 2).

A closer examination of the predictions made by the Mpipi-Recharged model reveals that, in addition to capturing the importance of cation–*π* and *π*–*π* contacts, it also identifies prevalent interactions involving aromatic residues (Y and F) and arginine with asparagine (N) and glutamine (Q) within A1-LCD condensates. These predictions are consistent with experimental phase diagrams of A1-LCD systems and align with the stickers-and-spacers framework, which demonstrates that tyrosine, phenylalanine, and tryptophan act as primary stickers, arginine serves as a context-dependent sticker, and other amino acids function as spacers [77, 117].

Recent advancements in residue-resolution coarse-grained models for biomolecular phase separation have led to significant progress. Beyond the examples presented here, models such as the HPS-Urry model, the CALVADOS family, and the Mpipi family have demonstrated exceptional predictive power in describing the relative thermodynamic stability of a wide range of biomolecular condensates, as well as single-molecule observables of proteins, in agreement with experimental data. Although the HPS model does not perform particularly well in our benchmarks, it is important to highlight that all other models were built upon the foundational framework provided by the pioneering HPS model [41, 52, 56]. Thus, it is unsurprising that these newer models outperform the original HPS model, reflecting the continuous progress in the field.

There are now many more sequence-dependent residue-resolution CG models, such as the original CALVADOS [41], KH [86] or the FB-HPS [58], which were not included in this work due to computational constraints, which limit the number of models feasible to simulate. However, as demonstrated in our previous study [53], these models perform reasonably well in capturing the phase diagrams of A1-LCD condensates and their mutants.

With this study, we aim to highlight the continuous advancements in residue-resolution coarse-grained model development, which is increasingly enabling the quantitative description of biomolecular phase behaviour while providing complementary microscopic insights that are often challenging to obtain through traditional experimental methods.

## 4 Methods

### 4.1 Model and methods

In our simulations, we represent the intrinsically disordered regions (IDRs) as coarse-grained structures with one bead per amino acid. The potential energy of the coarse-grained force field is defined as follows:

$$\begin{array}{c} E=E_{\text{Bonds}}+E_{\text{Electrostatic}}+E_{\text{Hydrophobic}}+E_{\text{cation-}\pi } \end{array}$$ (1)

In Table 1 we provide a summary of the different potentials used by each model studied in this work. To better visualize the differences in interactions between the different models, S1 Fig shows the normalised values of interaction strength for all amino acid pairs. Higher values indicate stronger attractive interactions, and lower values indicate weaker or repulsive interactions.

**Table 1:** Table of equations used by each model. The details of the equations are given below.

Energy	*E*_Bonds_	*E*_Electrostatic_	*E*_Hydrophobic_	*E*_cation-*π*_
Model				
HPS	(2)	(3)	(7)	-
HPS-cation-*π*	(2)	(3)	(7)	(10)
HPS-Urry	(2)	(3)	(7)	-
CALVADOS2	(2)	(3)	(7)	-
Mpipi	(2)	(3)	(8)	-
Mpipi-Recharged	(2)	(6)	(8)	-

Bonded interactions are modeled by a harmonic potential

$$\begin{array}{c} E_{\text{Bonds}}=\sum k{\left(r_{i}-r_{0}\right)}^{2}, \end{array}$$ (2)

where *k* = 9.6kJ/mol⋅Å^2^ for all the models except for the HPS-cation-*π* which is *k* = 2.4kJ/mol⋅Å^2^ and for the HPS-Urry which is *k* = 4.8kJ/mol⋅Å^2^. The equilibrium bond length is *r*_0_ = 3.81 Å between bonded amino acid beads.

The electrostatic interactions (*E*_Electrostatic_) among charged amino acids are governed by a Debye-Hückel potential

$$\begin{array}{c} E_{\text{Electrostatic}}=\sum_{i}\sum_{j<i}\frac{1}{4\pi \epsilon_{r}}\frac{q_{i}q_{j}}{r}e^{-\kappa r}, \end{array}$$ (3)

where *q*_*i*_ and *q*_*j*_ represent the charges of the beads *i* and *j*, *ϵ*_*r*_ = 80 *ϵ*_0_, is the relative dielectric constant of water (being *ϵ*_0_ the electric constant), *r* is the distance between the *i*th and *j*th beads, and *κ* = 1 nm^−1^ is the Debye screening length that mimics the implicit solvent (water and ions) at physiological salt concentration (*c*_*s*_∼150mM of NaCl) for the models HPS, HPS-cation-*π*, and the HPS-Urry model, and for the Mpipi model the value is *κ* = 1.26 nm^−1^. For the CALVADOS2 and Mpipi-Recharged model *κ* depends on the salt concentration as

$$\begin{array}{c} \kappa =\sqrt{8\pi Bc_{s}}, \end{array}$$ (4)

where *B*(*ϵ*_*r*_) = *e*^2^/4*πk*_*B*_*Tϵ*_0_*ϵ*_*r*_ is the Bjerrum length. The relative dielectric permittivity depends on the temperature according to this empirical law

$$\epsilon_{r}\left(T\right)=\frac{5321}{T}+233.76-0.9297T+1.417\cdot 10^{-3}T^{2}-8.292\cdot 10^{-7}T^{3}.$$ (5)

For the Mpipi-Recharged model, electrostatic interactions are modelled through a Yukawa potential given by

$$\begin{array}{c} E_{\text{Electrostatic}}=\sum_{i}\sum_{j<i}\frac{A_{ij}}{r}e^{-\kappa r}, \end{array}$$ (6)

where *A*_*ij*_ controls the interaction between a pair of amino acids and *κ* modulates the salt concentration in an explicit way. The dielectric constant varies with temperature according to Eqs (4) and (5).

For the HPS, HPS-cation-*π*, HPS-Urry and CALVADOS2 models, the hydrophobic interactions are established using a scale of hydrophobicity derived from statistical analysis of amino acid contacts in PDB structures. These interactions are incorporated using the Ashbaugh-Hatch potential [43, 86, 120, 121] functional form given by

$$E_{\text{Hydrophobic}}=\underset{i}{\sum }\underset{j<i}{\sum }\left\{\begin{array}{l} 4\epsilon_{ij}\left[{\left(\frac{\sigma_{ij}}{r}\right)}^{12}-{\left(\frac{\sigma_{ij}}{r}\right)}^{6}\right]+\left(1-\lambda_{ij}\right)\epsilon_{ij},\;\text{if}\;r<2^{1/6}\sigma_{ij},\\ \lambda_{ij}4\epsilon_{ij}\left[{\left(\frac{\sigma_{ij}}{r}\right)}^{12}-{\left(\frac{\sigma_{ij}}{r}\right)}^{6}\right],\;\text{otherwise}. \end{array}\right.$$ (7)

where λ_*i*_ and λ_*j*_ are parameters that account for the hydrophobicity of the *i*th and *j*th interacting particles respectively, being λ_*ij*_ = (λ_*i*_ + λ_*j*_)/2 following the Lorentz-Berthelot mixing rules [122, 123]. The excluded volume of the different residues is given by *σ*_*i*_ and *σ*_*j*_, where *σ*_*ij*_ = (*σ*_*i*_ + *σ*_*j*_)/2, and *r* is the distance between the *ij* particles. The parameter *ϵ*_*ij*_ is set to 0.2 kcal/mol to reproduce experimental single-IDR radius of gyration [43].

In the Mpipi and Mpipi-Recharged models, the hydrophobic interactions are parametrized by the Wang-Frenkel potential given by

$$\begin{array}{c} E_{\text{Hydrophobic}}=\sum_{i}\sum_{j<i}\epsilon_{ij}\alpha ({\left[\frac{\sigma_{ij}}{r}\right]}^{2\mu }-1){\left({\left[\frac{r_{c}}{r}\right]}^{2\mu }-1\right)}^{2\nu_{ij}} \end{array}$$ (8)

where

$$\begin{array}{c} \alpha =2\nu {\left(\frac{r_{c}}{\sigma_{ij}}\right)}^{2\mu }{\left[\frac{1+2\nu_{ij}}{2\nu_{ij}({\left(\frac{r_{c}}{\sigma_{ij}}\right)}^{2\mu }-1)}\right]}^{2\nu_{ij}+1}, \end{array}$$ (9)

where the excluded volume of the different residues is given by *σ*_*ij*_, *r* is the distance between the *ij* particles, *ϵ*_*ij*_ is the energy interaction parameter, *r*_*c*_ = 3*σ*_*ij*_ is the cut-off of the potential between those amino acids and *μ* = 1 and *ν*_*ij*_ are terms that are involved in the shape of the potential. The values of *σ*_*ij*_ and *ϵ*_*ij*_ are precisely parametrized for each interaction [61, 79]

For the HPS-cation-*π* model another additional component is added. A reparametrisation of the interactions between the positively charged amino acids and the aromatic ones as a Lennard-Jones potential is modelled as

$$\begin{array}{c} E_{\text{cation-}\pi }=4\epsilon_{ij}\left[{\left(\frac{\sigma_{ij}}{r}\right)}^{12}-{\left(\frac{\sigma_{ij}}{r}\right)}^{6}\right]. \end{array}$$ (10)

We employed a cut-off of 3*σ*_*ij*_ for the hydrophobic interactions, and 3.5 nm for the electrostatic ones [43], except for the HPS-Urry that uses a cut-off of 2.0 nm and for the CALVADOS2 that uses a cut-off of 4.0 nm [55].

### 4.2 Preparation of the systems and simulation details

All simulations were conducted using the LAMMPS software [124, 125]. Both Direct coexistence (DC) simulations and viscosity calculations were performed in the NVT ensemble employing a Langevin thermostat [126] with a relaxation time of 5 ps and a time step of 10 fs for the Verlet integration. The configurations for DC simulations were generated by placing 200 protein replicas in a slab with ∼17x17nm^2^ of section and 120nm of length for a resulting average density of ∼0.1 g/cm^3^. Simulations to calculate the coexistence densities run a total time of the order of ∼1*μ*s after reaching equilibrirum. Calculation of the viscosity was carried out from NVT simulations by placing 200 protein replicas in a cubic box that was isotropically compressed to reach the desired bulk density. After equilibration, production run took around ∼3 − 5*μ*s to fully capture the stress relaxation of each specific system.

We provide Supporting information and a GitHub Repository at https://github.com/Reshiiiii/hnRNPA1_Data_Scripts that contains the LAMMPS input scripts for every studied model as well as the configuration file for the considered A1-LCD mutants. We also provide sampling videos of representative trajectories concerning the direct coexistence method, viscosity and different conformations of a protein.

### 4.3 Direct Coexistence technique

Employing Direct Coexistence (DC) simulations [84, 127–129], we determined the phase diagram for each of the studied proteins [78]. This method involves simulating the two coexisting phases within the same simulation box. In our approach, we arranged a high-density protein liquid alongside a very low-density counterpart. To accommodate the diverse densities, we employed an elongated simulation box, therefore allowing for the formation of both coexisting phases forming an interface perpendicular to the long direction of the simulation box. Equilibrium was attained through NVT simulations, and subsequently, we measured the equilibrium coexisting densities of both phases along the elongated side of the box. This process was repeated across various temperatures until reaching the critical temperature. To mitigate finite system-size effects near the critical point, we calculated the critical temperature (*T*_c_) and density (*ρ*_c_) using the law of critical exponents and rectilinear diameters [130]

$$\begin{array}{c} {\left(\rho_{l}-\rho_{v}\right)}^{\alpha }=s_{1}(1-\frac{T}{T_{c}}) \end{array}$$ (11)

and

$$\begin{array}{c} \frac{\rho_{l}+\rho_{v}}{2}=\rho_{c}+s_{2}\left(T_{c}-T\right), \end{array}$$ (12)

where the notation *ρ*_*l*_ and *ρ*_*v*_ are the densities of the condensed and diluted phases, respectively. Moreover, s_1_ and s_2_ are fitting parameters, while the critical exponent, *α* = 3.06 for the three-dimensional Ising model [130]. The critical temperature error has been determined using the law of critical exponents and rectilinear diameters to estimate the system’s critical temperature by considering the last two, three, and four temperatures.

### 4.4 Calculating contact maps and top contacts

The computation of intermolecular and intramolecular contact maps within protein condensates is calculated from DC trajectories. Contacts were determined across all systems at temperatures approximately 0.95*T*_c_, where *T*_*c*_ corresponds to the critical temperature of each system. Typically, molecular contacts are identified based on a distance criterion, with the assumption that the relative frequency of contact map occurrences (rather than absolute frequency) remains generally unaffected by the selected cut-off distance used in calculations, provided the cut-off values are reasonable. However, to accurately capture the most relevant and common residue-residue contact pairs that facilitate LLPS, it is highly recommended to account for the specific parametrized of each amino acid in terms of excluded volume and minimum potential energy interaction distance. Hence, we adopted a sequence-dependent cut-off distance equivalent to 1.2*σ*_*ij*_, where *σ*_*ij*_ represents the mean excluded volume of the respective ith and jth amino acids [69]. Given that the minimum of the potential used lies at approximately 2^1/6^*σ*_*ij*_ ≈ 1.122*σ*_*ij*_, we set the cut-off distance slightly beyond this point, at 1.2*σ*_*ij*_, to ensure significant binding. By implementing this innovative sequence-dependent cut-off scheme for each amino acid pair interaction, we can effectively filter out adjacent contacts that may coincide with actual interacting amino acids along the sequence, thus enhancing our ability to accurately identify the amino acids that positively contribute to stabilising condensates [69].

To define the relative interaction strength for the most relevant contacts, this interaction frequency described above (*f*_i,j_) is weighted with the hydrophobic and electrostatic contributions of each amino acid pair for the model ($E_{ij}^{\text{Hydrophobic}}+E^{\text{Electrostatic}}$), normalised against the occurrence frequency of each pair of amino acids in the sequence, as described by

$$Interaction\;strength=f_{ij}\frac{E_{ij}^{\text{Hydrophobic}}+E_{ij}^{\text{Electrostatic}}}{N_{i}N_{j}}.$$ (13)

### 4.5 Calculating viscosities

We can determine the viscosity of the LCD model’s condensate using the Green-Kubo (GK) relation. The time-dependent mechanical response of a viscoelastic material under small shear deformation is characterized by the shear stress relaxation modulus (*G*(*t*)) [131]. When subjected to zero deformation, *G*(*t*) can be computed by correlating any off-diagonal component of the pressure tensor at equilibrium [132, 133]


$$\begin{array}{c} G\left(t\right)=\frac{V}{k_{B}T}\left\langle \sigma_{\alpha \beta }\left(t\right)\sigma_{\alpha \beta }\left(0\right)\right\rangle . \end{array}$$ (14)

In Eq 14, *σ*_*α*,*β*_ represents an off-diagonal component of the stress tensor, *V* is the volume, *T* is the system temperature and *k*_*B*_ is the Boltzmann constant. For isotropic systems, a more precise expression of *G*(*t*) is obtained by considering the six independent components of the pressure tensor [110, 112, 134, 135], given by

$$\begin{array}{ll} G\left(t\right)= & \frac{V}{5k_{B}T}[\langle \sigma_{xy}(t)\sigma_{xy}(0)\rangle +\langle \sigma_{xz}(t)\sigma_{xz}(0)\rangle +\langle \sigma_{yz}(t)\sigma_{yz}(0)\rangle \\ & +\frac{1}{6}(\langle N_{xy}(t)N_{xy}(0)\rangle +\langle N_{xz}(t)N_{xz}(0)\rangle +\langle N_{yz}(t)N_{yz}(0)\rangle )], \end{array}$$ (15)

where *N*_*α*,*β*_ = *σ*_*α*,*α*_ − *σ*_*β*,*β*_ represents the normal stress difference. Once the relaxation modulus is obtained, shear viscosity (*η*) is calculated by integrating the shear stress relaxation modulus over time using the last GK formula

$$\begin{array}{c} \eta =\int_{0}^{\infty }G\left(t\right)\;dt. \end{array}$$ (16)

To mitigate noise in the relaxation modulus observed in protein condensate simulations [31, 69], we adopt a specific strategy for viscosity estimation. While *G*(*t*) is smooth at short timescales so that it can be numerically integrated, at longer timescales *G*(*t*) is fitted to a series of Maxwell modes (*G*_*i*_ exp(-*t*/*τ*_i_)) equidistant in logarithmic time, and then analytically integrated. Thus, viscosity is calculated by combining two terms as

$$\begin{array}{c} \eta =\eta \left(t_{0}\right)+\sum_{i=1}^{M}G_{i}\tau_{i}, \end{array}$$ (17)

where *η*(*t*_0_) represents the term computed at short times by trapezoidal integration, $\sum_{i=1}^{M}G_{i}\tau_{i}$ corresponds to the part evaluated via the Maxwell modes fit at long timescales, and *t*_0_ denotes the time after which all intramolecular oscillations of *G*(*t*) have decayed and the function becomes strictly positive and decays monotonically.

### 4.6 Deviation from the ideal line in interpolations

For cases where simulated saturation concentration have been plotted against experimental values (Fig 5 in the main text and S2 Fig), the following formula has been used to calculate the deviation of simulated results from experimental ones

$$\begin{array}{c} D=\frac{1}{n}\sum_{i=1}^{n}\frac{\left|\text{log}\left(x_{\text{exp},i}\right)-\text{log}\left(x_{\text{sim},i}\right)\right|}{\text{log}(x_{\text{exp},i})}, \end{array}$$ (18)

where log represents the decimal logarithm.

For cases where simulated viscosity has been plotted against experimental values (Fig 6) the following formula has been used to calculate the deviation of simulated results from experimental ones

$$\begin{array}{c} D=\frac{1}{n}\sum_{i=1}^{n}{\left(\text{log}\left(x_{\text{exp},i}\right)-\text{log}\left(x_{\text{sim},i}\right)\right)}^{2}, \end{array}$$ (19)

where log represents the decimal logarithm.

## Supporting information

S1 Table Amino acid sequences of the different hnRNPA1-LCD variants. The amino acid sequences of the hnRNPA1-LCD variants utilized in this investigation. In the first column, we have listed the names of the variants. The second column displays their amino acid sequences, while the third column indicates the number and type of changes compared to the wild-type sequence.
(EPS)

S1 Fig Relative interaction strength values (including electrostatic + hydrophobic interactions) for the HPS (A), HPS-cation-*π* (B), HPS-Urry (C), CALVADOS2 (D), Mpipi (E), and Mpipi-Recharged (F) models. The values have been normalised by the highest interaction value of each model.
(EPS)

S2 Fig Simulated vs. experimental saturation concentration [78] for the different variants and for the models CALVADOS2 (A), Mpipi (B) and Mpipi-Recharged (C). The Pearson correlation coefficient (*r*), the slope (*m*), and the root mean square deviation from the experimental values (*D*) are displayed for each set of modelling data. The black lines indicate a perfect match between experimental and computational values, while the red dashed lines depict the linear regression for each set of data.
(EPS)

S3 Fig Contact maps of potential energy interaction for the A1-LCD (WT+NLS) sequence. HPS (A), HPS-cation-*π* (B), HPS-Urry (C), CALVADOS2 (D), Mpipi (E), and Mpipi-Recharged (F) models at T = 0.95 T_*c*_ (where T_*c*_ refers to the critical temperature of the WT+NLS sequence of each model) and at the condensate equilibrium density corresponding to such temperature. The associated energy corresponding to a given specific interaction is depicted by the side bar. Details on these calculations are provided in the Methods Section.
(EPS)

S4 Fig Predominant intermolecular interactions contributing to LLPS of A1-LCD (WT+NLS) protein. HPS (A), HPS-cation-*π* (B), HPS-Urry (C), CALVADOS2 (D), and Mpipi (E) models. The presented contribution by each residue-residue pair has been normalised by the highest contact pair. Moreover, normalisation by the pair residue-residue abundance across the sequence has been applied as detailed in the Methods Section.
(EPS)

S1 Video Simulation movie of a representative trajectory of A1-LCD using the Direct Coexistence method to compute the phase diagram. (MP4)

S2 Video Simulation movie of a representative trajectory of A1-LCD performed for the calculation of the condensate viscosity. (MP4)

## References

[1] Hyman AA, Simons K. Beyond Oil and Water-Phase Transitions in Cells. Science. 2012;337(6098):1047–1049. doi: 10.1126/science.1223728 22936764

[2] Hyman AA, Weber CA, Jülicher F. Liquid-liquid phase separation in biology. Annual review of cell and developmental biology. 2014;30:39–58. doi: 10.1146/annurev-cellbio-100913-013325 25288112

[3] Brangwynne CP, Eckmann CR, Courson DS, Rybarska A, Hoege C, Gharakhani J, et al. Germline P granules are liquid droplets that localize by controlled dissolution/condensation. Science. 2009;324(5935):1729–1732. doi: 10.1126/science.1172046 19460965

[4] Su X, Ditlev JA, Hui E, Xing W, Banjade S, Okrut J, et al. Phase separation of signaling molecules promotes T cell receptor signal transduction. Science. 2016;352(6285):595–599. doi: 10.1126/science.aad9964 27056844 PMC4892427

[5] Su Q, Mehta S, Zhang J. Liquid-liquid phase separation: Orchestrating cell signaling through time and space. Molecular cell. 2021;81(20):4137–4146. doi: 10.1016/j.molcel.2021.09.010 34619090 PMC8541918

[6] Xiao Q, McAtee CK, Su X. Phase separation in immune signalling. Nature Reviews Immunology. 2022;22(3):188–199. doi: 10.1038/s41577-021-00572-5 34230650 PMC9674404

[7] Boeynaems S, Alberti S, Fawzi NL, Mittag T, Polymenidou M, Rousseau F, et al. Protein phase separation: a new phase in cell biology. Trends in cell biology. 2018;28(6):420–435. doi: 10.1016/j.tcb.2018.02.004 29602697 PMC6034118

[8] Zhou H, Song Z, Zhong S, Zuo L, Qi Z, Qu LJ, et al. Mechanism of DNA-induced phase separation for transcriptional repressor VRN1. Angewandte Chemie. 2019;131(15):4912–4916. doi: 10.1002/ange.201810373 30762296

[9] Lemkul JA, Bevan DR. Assessing the stability of Alzheimer’s amyloid protofibrils using molecular dynamics. The Journal of Physical Chemistry B. 2010;114(4):1652–1660. doi: 10.1021/jp9110794 20055378

[10] Musacchio A. On the role of phase separation in the biogenesis of membraneless compartments. The EMBO journal. 2022;41(5):e109952. doi: 10.15252/embj.2021109952 35107832 PMC8886532

[11] Strom AR, Emelyanov AV, Mir M, Fyodorov DV, Darzacq X, Karpen GH. Phase separation drives heterochromatin domain formation. Nature. 2017;547(7662):241–245. doi: 10.1038/nature22989 28636597 PMC6022742

[12] Narlikar GJ. Phase-separation in chromatin organization. Journal of Biosciences. 2020;45(1):5. doi: 10.1007/s12038-019-9978-z 31965983 PMC9107952

[13] Deviri D, Safran SA. Physical theory of biological noise buffering by multicomponent phase separation. Proceedings of the National Academy of Sciences. 2021;118(25):e2100099118. doi: 10.1073/pnas.2100099118 34135122 PMC8237649

[14] Riback JA, Brangwynne CP. Can phase separation buffer cellular noise? Science. 2020;367(6476):364–365. 31974233 10.1126/science.aba0446

[15] Klosin A, Oltsch F, Harmon T, Honigmann A, Jülicher F, Hyman AA, et al. Phase separation provides a mechanism to reduce noise in cells. Science. 2020;367(6476):464–468. doi: 10.1126/science.aav6691 31974256

[16] Sheu-Gruttadauria J, MacRae IJ. Phase transitions in the assembly and function of human miRISC. Cell. 2018;173(4):946–957. doi: 10.1016/j.cell.2018.02.051 29576456 PMC5935535

[17] Bouchard JJ, Otero JH, Scott DC, Szulc E, Martin EW, Sabri N, et al. Cancer mutations of the tumor suppressor SPOP disrupt the formation of active, phase-separated compartments. Molecular cell. 2018;72(1):19–36. doi: 10.1016/j.molcel.2018.08.027 30244836 PMC6179159

[18] Qamar S, Wang G, Randle SJ, Ruggeri FS, Varela JA, Lin JQ, et al. FUS phase separation is modulated by a molecular chaperone and methylation of arginine cation-*π* interactions. Cell. 2018;173(3):720–734. doi: 10.1016/j.cell.2018.03.056 29677515 PMC5927716

[19] Murthy AC, Dignon GL, Kan Y, Zerze GH, Parekh SH, Mittal J, et al. Molecular interactions underlying liquid- liquid phase separation of the FUS low-complexity domain. Nature structural & molecular biology. 2019;26(7):637–648. doi: 10.1038/s41594-019-0250-x 31270472 PMC6613800

[20] Carey JL, Guo L. Liquid-liquid phase separation of TDP-43 and FUS in physiology and pathology of neurodegenerative diseases. Frontiers in molecular biosciences. 2022;9:826719. doi: 10.3389/fmolb.2022.826719 35187086 PMC8847598

[21] Li HR, Chiang WC, Chou PC, Wang WJ, Huang Jr. TAR DNA-binding protein 43 (TDP-43) liquid–liquid phase separation is mediated by just a few aromatic residues. Journal of Biological Chemistry. 2018;293(16):6090–6098. doi: 10.1074/jbc.AC117.001037 29511089 PMC5912450

[22] McGurk L, Gomes E, Guo L, Mojsilovic-Petrovic J, Tran V, Kalb RG, et al. Poly (ADP-ribose) prevents pathological phase separation of TDP-43 by promoting liquid demixing and stress granule localization. Molecular cell. 2018;71(5):703–717. doi: 10.1016/j.molcel.2018.07.002 30100264 PMC6128762

[23] Martin EW, Thomasen FE, Milkovic NM, Cuneo MJ, Grace CR, Nourse A, et al. Interplay of folded domains and the disordered low-complexity domain in mediating hnRNPA1 phase separation. Nucleic acids research. 2021;49(5):2931–2945. doi: 10.1093/nar/gkab063 33577679 PMC7969017

[24] Molliex A, Temirov J, Lee J, Coughlin M, Kanagaraj AP, Kim HJ, et al. Phase separation by low complexity domains promotes stress granule assembly and drives pathological fibrillization. Cell. 2015;163(1):123–133. doi: 10.1016/j.cell.2015.09.015 26406374 PMC5149108

[25] Guo L, Shorter J. It’s raining liquids: RNA tunes viscoelasticity and dynamics of membraneless organelles. Molecular cell. 2015;60(2):189–192. doi: 10.1016/j.molcel.2015.10.006 26474062 PMC4653132

[26] Wang X, Schwartz JC, Cech TR. Nucleic acid-binding specificity of human FUS protein. Nucleic acids research. 2015;43(15):7535–7543. Available from: http://www.ncbi.nlm.nih.gov/pubmed/26150427 http://www.pubmedcentral.nih.gov/articlerender.fcgi?artid=PMC4551922. doi: 10.1093/nar/gkv679 26150427 PMC4551922

[27] Farag M, Cohen SR, Borcherds WM, Bremer A, Mittag T, Pappu RV. Condensates formed by prion-like low-complexity domains have small-world network structures and interfaces defined by expanded conformations. Nature communications. 2022;13(1):7722. doi: 10.1038/s41467-022-35370-7 36513655 PMC9748015

[28] Borcherds W, Bremer A, Borgia MB, Mittag T. How do intrinsically disordered protein regions encode a driving force for liquid–liquid phase separation? Current opinion in structural biology. 2021;67:41–50. doi: 10.1016/j.sbi.2020.09.004 33069007 PMC8044266

[29] Hughes MP, Sawaya MR, Boyer DR, Goldschmidt L, Rodriguez JA, Cascio D, et al. Atomic structures of low-complexity protein segments reveal kinked *β* sheets that assemble networks. Science. 2018;359(6376):698–701. doi: 10.1126/science.aan6398 29439243 PMC6192703

[30] Ray S, Singh N, Kumar R, Patel K, Pandey S, Datta D, et al. *α*-Synuclein aggregation nucleates through liquid–liquid phase separation. Nature chemistry. 2020;12(8):705–716. doi: 10.1038/s41557-020-0465-9 32514159

[31] Tejedor AR, Sanchez-Burgos I, Estevez-Espinosa M, Garaizar A, Collepardo-Guevara R, Ramirez J, et al. Protein structural transitions critically transform the network connectivity and viscoelasticity of RNA-binding protein condensates but RNA can prevent it. Nature communications. 2022;13(1):1–15. doi: 10.1038/s41467-022-32874-0 PMC9522849 36175408

[32] Gopal PP, Nirschl JJ, Klinman E, Holzbaur EL. Amyotrophic lateral sclerosis-linked mutations increase the viscosity of liquid-like TDP-43 RNP granules in neurons. Proceedings of the National Academy of Sciences. 2017;114(12):E2466–E2475. doi: 10.1073/pnas.1614462114 28265061 PMC5373408

[33] St George-Hyslop P, Lin JQ, Miyashita A, Phillips EC, Qamar S, Randle SJ, et al. The physiological and pathological biophysics of phase separation and gelation of RNA binding proteins in amyotrophic lateral sclerosis and fronto-temporal lobar degeneration. Brain research. 2018;1693:11–23. doi: 10.1016/j.brainres.2018.04.036 29723523 PMC6018615

[34] Patel A, Lee HO, Jawerth L, Maharana S, Jahnel M, Hein MY, et al. A liquid-to-solid phase transition of the ALS protein FUS accelerated by disease mutation. Cell. 2015;162(5):1066–1077. doi: 10.1016/j.cell.2015.07.047 26317470

[35] Mackenzie IR, Nicholson AM, Sarkar M, Messing J, Purice MD, Pottier C, et al. TIA1 mutations in amyotrophic lateral sclerosis and frontotemporal dementia promote phase separation and alter stress granule dynamics. Neuron. 2017;95(4):808–816. doi: 10.1016/j.neuron.2017.07.025 28817800 PMC5576574

[36] Lu Y, Lim L, Song J. RRM domain of ALS/FTD-causing FUS characteristic of irreversible unfolding spontaneously self-assembles into amyloid fibrils. Scientific Reports. 2017;7(1):1–14. doi: 10.1038/s41598-017-01281-7 28432364 PMC5430844

[37] Ambadipudi S, Biernat J, Riedel D, Mandelkow E, Zweckstetter M. Liquid–liquid phase separation of the microtubule-binding repeats of the Alzheimer-related protein Tau. Nature communications. 2017;8(1):1–13. doi: 10.1038/s41467-017-00480-0 28819146 PMC5561136

[38] Bishof I, Dammer EB, Duong DM, Kundinger SR, Gearing M, Lah JJ, et al. RNA-binding proteins with basic-acidic dipeptide (BAD) domains self-assemble and aggregate in Alzheimer’s disease. Journal of Biological Chemistry. 2018;293(28):11047–11066. doi: 10.1074/jbc.RA118.001747 29802200 PMC6052236

[39] Spannl S, Tereshchenko M, Mastromarco GJ, Ihn SJ, Lee HO. Biomolecular condensates in neurodegeneration and cancer. Traffic. 2019;20(12):890–911. doi: 10.1111/tra.12704 31606941

[40] Mehta S, Zhang J. Liquid–liquid phase separation drives cellular function and dysfunction in cancer. Nature Reviews Cancer. 2022;22(4):239–252. doi: 10.1038/s41568-022-00444-7 35149762 PMC10036213

[41] Tesei G, Schulze TK, Crehuet R, Lindorff-Larsen K. Accurate model of liquid–liquid phase behavior of intrinsically disordered proteins from optimization of single-chain properties. Proceedings of the National Academy of Sciences. 2021;118(44):e2111696118. doi: 10.1073/pnas.2111696118 34716273 PMC8612223

[42] Szała-Mendyk B, Phan TM, Mohanty P, Mittal J. Challenges in studying the liquid-to-solid phase transitions of proteins using computer simulations. Current Opinion in Chemical Biology. 2023;75:102333. doi: 10.1016/j.cbpa.2023.102333 37267850 PMC10527940

[43] Dignon GL, Zheng W, Kim YC, Best RB, Mittal J. Sequence determinants of protein phase behavior from a coarse-grained model. PLoS computational biology. 2018;14(1):e1005941. doi: 10.1371/journal.pcbi.1005941 29364893 PMC5798848

[44] Paloni M, Bailly R, Ciandrini L, Barducci A. Unraveling molecular interactions in liquid–liquid phase separation of disordered proteins by atomistic simulations. The Journal of Physical Chemistry B. 2020;124(41):9009–9016. doi: 10.1021/acs.jpcb.0c06288 32936641

[45] Zheng W, Dignon GL, Jovic N, Xu X, Regy RM, Fawzi NL, et al. Molecular details of protein condensates probed by microsecond long atomistic simulations. The Journal of Physical Chemistry B. 2020;124(51):11671–11679. doi: 10.1021/acs.jpcb.0c10489 33302617 PMC7879053

[46] Sponer J, Krepl M, Banas P, Kuhrova P, Zgarbova M, Jurecka P, et al. How to understand atomistic molecular dynamics simulations of RNA and protein–RNA complexes? Wiley Interdisciplinary Reviews: RNA. 2017;8(3):e1405. doi: 10.1002/wrna.1405 27863061

[47] Shim HS, Iaconelli J, Shang X, Li J, Lan ZD, Jiang S, et al. TERT activation targets DNA methylation and multiple aging hallmarks. Cell. 2024; 187(15): p4030–4042.e13. doi: 10.1016/j.cell.2024.10.048 38908367 PMC11552617

[48] Garaizar A, Higginbotham T, Sanchez-Burgos I, Tejedor AR, Sanz E, Espinosa JR. Alternating one-phase and two-phase crystallization mechanisms in octahedral patchy colloids. The Journal of Chemical Physics. 2022;157(13). doi: 10.1063/5.0101529 36209006

[49] Espinosa JR, Garaizar A, Vega C, Frenkel D, Collepardo-Guevara R. Breakdown of the law of rectilinear diameter and related surprises in the liquid-vapor coexistence in systems of patchy particles. The Journal of chemical physics. 2019;150(22):224510. doi: 10.1063/1.5098551 31202247 PMC6626546

[50] Nguemaha V, Zhou HX. Liquid-liquid phase separation of patchy particles illuminates diverse effects of regulatory components on protein droplet formation. Scientific reports. 2018;8(1):1–11. doi: 10.1038/s41598-018-25132-1 29712961 PMC5928213

[51] Sanchez-Burgos I, Espinosa JR, Joseph JA, Collepardo-Guevara R. Valency and Binding Affinity Variations Can Regulate the Multilayered Organization of Protein Condensates with Many Components. Biomolecules. 2021;11(2):278. doi: 10.3390/biom11020278 33672806 PMC7918469

[52] Dignon GL, Zheng W, Kim YC, Best RB, Mittal J. Sequence determinants of protein phase behavior from a coarse-grained model. PLoS computational biology. 2018;14(1):e1005941. doi: 10.1371/journal.pcbi.1005941 29364893 PMC5798848

[53] Joseph JA, Reinhardt A, Aguirre A, Chew PY, Russell KO, Espinosa JR, et al. Physics-driven coarse-grained model for biomolecular phase separation with near-quantitative accuracy. Nature Computational Science. 2021;1(11):732–743. doi: 10.1038/s43588-021-00155-3 35795820 PMC7612994

[54] Regy RM, Thompson J, Kim YC, Mittal J. Improved coarse-grained model for studying sequence dependent phase separation of disordered proteins. Protein Science. 2021;30(7):1371–1379. doi: 10.1002/pro.4094 33934416 PMC8197430

[55] Tesei G, Lindorff-Larsen K. Improved predictions of phase behaviour of intrinsically disordered proteins by tuning the interaction range. Open Research Europe. 2023;2:94. doi: 10.12688/openreseurope.14967.2 37645312 PMC10450847

[56] Das S, Lin YH, Vernon RM, Forman-Kay JD, Chan HS. Comparative roles of charge, *π*, and hydrophobic interactions in sequence-dependent phase separation of intrinsically disordered proteins. Proceedings of the National Academy of Sciences. 2020;117(46):28795–28805. doi: 10.1073/pnas.2008122117 33139563 PMC7682375

[57] Lotthammer JM, Ginell GM, Griffith D, Emenecker R, Holehouse AS. Direct prediction of intrinsically disordered protein conformational properties from sequence. Biophysical Journal. 2024;123(3):43a. doi: 10.1038/s41592-023-02159-5 38297184 PMC10927563

[58] Dannenhoffer-Lafage T, Best RB. A data-driven hydrophobicity scale for predicting liquid–liquid phase separation of proteins. The Journal of Physical Chemistry B. 2021;125(16):4046–4056. doi: 10.1021/acs.jpcb.0c11479 33876938 PMC12442143

[59] Garaizar A, Espinosa JR. Salt dependent phase behavior of intrinsically disordered proteins from a coarse-grained model with explicit water and ions. The Journal of Chemical Physics. 2021;155(12):125103. doi: 10.1063/5.0062687 34598583

[60] Statt A, Casademunt H, Brangwynne CP, Panagiotopoulos AZ. Model for disordered proteins with strongly sequence-dependent liquid phase behavior. The Journal of Chemical Physics. 2020;152(7):075101. doi: 10.1063/1.5141095 32087632

[61] Tejedor AR, Aguirre Gonzalez A, Maristany MJ, Chew PY, Rusell K, Ramirez J, et al. Chemically-informed coarse-graining of electrostatic forces in charge-rich biomolecular condensates. bioRxiv. 2024; https://www.biorxiv.org/content/early/2024/07/27/2024.07.26.605370. doi: 10.1021/acscentsci.4c01617 PMC11869137 40028356

[62] Murthy AC, Dignon GL, Kan Y, Zerze GH, Parekh SH, Mittal J, et al. Molecular interactions underlying liquid- liquid phase separation of the FUS low-complexity domain. Nature structural & molecular biology. 2019;26(7):637–648. doi: 10.1038/s41594-019-0250-x 31270472 PMC6613800

[63] Dignon GL, Best RB, Mittal J. Biomolecular Phase Separation: From Molecular Driving Forces to Macroscopic Properties. Annual Review of Physical Chemistry. 2020;71:53–75. doi: 10.1146/annurev-physchem-071819-113553 32312191 PMC7469089

[64] Dignon GL, Zheng W, Mittal J. Simulation methods for liquid–liquid phase separation of disordered proteins. Current opinion in chemical engineering. 2019;23:92–98. doi: 10.1016/j.coche.2019.03.004 32802734 PMC7426017

[65] Schuster BS, Dignon GL, Tang WS, Kelley FM, Ranganath AK, Jahnke CN, et al. Identifying sequence perturbations to an intrinsically disordered protein that determine its phase-separation behavior. Proceedings of the National Academy of Sciences. 2020;117(21):11421–11431. doi: 10.1073/pnas.2000223117 PMC7261017 32393642

[66] Ryan VH, Dignon GL, Zerze GH, Chabata CV, Silva R, Conicella AE, et al. Mechanistic view of hnRNPA2 low-complexity domain structure, interactions, and phase separation altered by mutation and arginine methylation. Molecular cell. 2018;69(3):465–479. doi: 10.1016/j.molcel.2017.12.022 29358076 PMC5801700

[67] Abyzov A, Blackledge M, Zweckstetter M. Conformational dynamics of intrinsically disordered proteins regulate biomolecular condensate chemistry. Chemical Reviews. 2022;122(6):6719–6748. doi: 10.1021/acs.chemrev.1c00774 35179885 PMC8949871

[68] Maristany M Julia, Aguirre Gonzalez Anne, Espinosa Jorge R, Huertas Jan, Collepardo-Guevara Rosana, Joseph Jerelle A (2024) Decoding Phase Separation of Prion-Like Domains through Data-Driven Scaling Laws eLife 13:RP99068 doi: 10.7554/eLife.99068.2 PMC11820118 39937084

[69] Tejedor AR, Garaizar A, Ramírez J, Espinosa JR. ‘RNA modulation of transport properties and stability in phase-separated condensates. Biophysical Journal. 2021;120(23):5169–5186. doi: 10.1016/j.bpj.2021.11.003 34762868 PMC8715277

[70] Sanchez-Burgos I, Espinosa JR, Joseph JA, Collepardo-Guevara R. RNA length has a non-trivial effect in the stability of biomolecular condensates formed by RNA-binding proteins. PLoS computational biology. 2022;18(2):e1009810. doi: 10.1371/journal.pcbi.1009810 35108264 PMC8896709

[71] Funari R, Bhalla N, Gentile L. Measuring the radius of gyration and intrinsic flexibility of viral proteins in buffer solution using small-angle X-ray Scattering. ACS Measurement Science Au. 2022;2(6):547–552. doi: 10.1021/acsmeasuresciau.2c00048 36573077 PMC9783065

[72] Huang X, Powers R. Validity of using the radius of gyration as a restraint in NMR protein structure determination. Journal of the American Chemical Society. 2001;123(16):3834–3835. doi: 10.1021/ja005770p 11457122

[73] Araki K, Yagi N, Nakatani R, Sekiguchi H, So M, Yagi H, et al. A small-angle X-ray scattering study of alpha-synuclein from human red blood cells. Scientific reports. 2016;6(1):30473. doi: 10.1038/srep30473 27469540 PMC4965831

[74] Riback JA, Bowman MA, Zmyslowski AM, Knoverek CR, Jumper JM, Hinshaw JR, et al. Innovative scattering analysis shows that hydrophobic disordered proteins are expanded in water. Science. 2017;358(6360):238–241. doi: 10.1126/science.aan5774 29026044 PMC5959285

[75] Gomes GNW, Krzeminski M, Namini A, Martin EW, Mittag T, Head-Gordon T, et al. Conformational ensembles of an intrinsically disordered protein consistent with NMR, SAXS, and single-molecule FRET. Journal of the American Chemical Society. 2020;142(37):15697–15710. doi: 10.1021/jacs.0c02088 32840111 PMC9987321

[76] Dignon GL, Zheng W, Best RB, Kim YC, Mittal J. Relation between single-molecule properties and phase behavior of intrinsically disordered proteins. Proceedings of the National Academy of Sciences. 2018;115(40):9929–9934. doi: 10.1073/pnas.1804177115 30217894 PMC6176625

[77] Bremer A, Farag M, Borcherds WM, Peran I, Martin EW, Pappu RV, et al. Deciphering how naturally occurring sequence features impact the phase behaviours of disordered prion-like domains. Nature Chemistry. 2022;14(2):196–207. doi: 10.1038/s41557-021-00840-w 34931046 PMC8818026

[78] Alshareedah I, Borcherds WM, Cohen SR, Singh A, Posey AE, Farag M, et al. Sequence-specific interactions determine viscoelasticity and ageing dynamics of protein condensates. Nature Physics. 2024;p. 1–10. doi: 10.1038/s41567-024-02558-1 39464253 PMC11501078

[79] Joseph JA, Reinhardt A, Aguirre A, Chew PY, Russell KO, Espinosa JR, et al. Physics-driven coarse-grained model for biomolecular phase separation with near-quantitative accuracy. Nature Computational Science. 2021;1(11):732–743. doi: 10.1038/s43588-021-00155-3 35795820 PMC7612994

[80] Li P, Banjade S, Cheng HC, Kim S, Chen B, Guo L, et al. Phase transitions in the assembly of multivalent signalling proteins. Nature. 2012;483(7389):336–340. doi: 10.1038/nature10879 22398450 PMC3343696

[81] Boeynaems S, Alberti S, Fawzi NL, Mittag T, Polymenidou M, Rousseau F, et al. Protein phase separation: a new phase in cell biology. Trends in cell biology. 2018;28(6):420–435. doi: 10.1016/j.tcb.2018.02.004 29602697 PMC6034118

[82] Vernon RM, Chong PA, Tsang B, Kim TH, Bah A, Farber P, et al. Pi-Pi contacts are an overlooked protein feature relevant to phase separation. elife. 2018;7:e31486. doi: 10.7554/eLife.31486 29424691 PMC5847340

[83] Martin EW, Holehouse AS, Peran I, Farag M, Incicco JJ, Bremer A, et al. Valence and patterning of aromatic residues determine the phase behavior of prion-like domains. Science. 2020;367(6478):694–699. doi: 10.1126/science.aaw8653 32029630 PMC7297187

[84] Ladd A, Woodcock L. Triple-point coexistence properties of the Lennard-Jones system. Chemical Physics Letters. 1977;51(1):155–159. doi: 10.1016/0009-2614(77)85375-X

[85] Espinosa JR, Joseph JA, Sanchez-Burgos I, Garaizar A, Frenkel D, Collepardo-Guevara R. Liquid network connectivity regulates the stability and composition of biomolecular condensates with many components. Proceedings of the National Academy of Sciences. 2020;117(24):13238–13247. doi: 10.1073/pnas.1917569117 32482873 PMC7306995

[86] Kapcha LH, Rossky PJ. A simple atomic-level hydrophobicity scale reveals protein interfacial structure. Journal of molecular biology. 2014;426(2):484–498. doi: 10.1016/j.jmb.2013.09.039 24120937

[87] Wessén J, Das S, Pal T, Chan HS. Analytical formulation and field-theoretic simulation of sequence-specific phase separation of protein-like heteropolymers with short-and long-spatial-range interactions. The Journal of Physical Chemistry B. 2022;126(45):9222–9245. doi: 10.1021/acs.jpcb.2c06181 36343363

[88] Urry DW, Gowda DC, Parker TM, Luan CH, Reid MC, Harris CM, et al. Hydrophobicity scale for proteins based on inverse temperature transitions. Biopolymers: Original Research on Biomolecules. 1992;32(9):1243–1250. doi: 10.1002/bip.360320913 1420991

[89] Feito A, Sanchez-Burgos I, Rey A, Collepardo-Guevara R, Espinosa JR, Tejedor AR. Capturing single-molecule properties does not ensure accurate prediction of biomolecular phase diagrams. Molecular Physics. 2024;p. e2425757. doi: 10.1080/00268976.2024.2425757

[90] Ladd A, Woodcock L. Triple-point coexistence properties of the Lennard-Jones system. Chemical Physics Letters. 1977;51(1):155–159. doi: 10.1016/0009-2614(77)85375-X

[91] Pal T, Wessén J, Das S, Chan HS. Differential Effects of Sequence-Local versus Nonlocal Charge Patterns on Phase Separation and Conformational Dimensions of Polyampholytes as Model Intrinsically Disordered Proteins. The Journal of Physical Chemistry Letters. 2024;15(32):8248–8256. doi: 10.1021/acs.jpclett.4c01973 39105804

[92] Alberti S, Dormann D. Liquid-liquid phase separation in disease. Annu Rev Genet. 2019;53(1):171–194. doi: 10.1146/annurev-genet-112618-043527 31430179

[93] Wei MT, Elbaum-Garfinkle S, Holehouse AS, Chen CCH, Feric M, Arnold CB, et al. Phase behaviour of disordered proteins underlying low density and high permeability of liquid organelles. Nature Chemistry. 2017;9(11):1118. doi: 10.1038/nchem.2803 29064502 PMC9719604

[94] Wang A, Conicella AE, Schmidt HB, Martin EW, Rhoads SN, Reeb AN, et al. A single N-terminal phosphomimic disrupts TDP-43 polymerization, phase separation, and RNA splicing. The EMBO journal. 2018;37(5):e97452. doi: 10.15252/embj.201797452 29438978 PMC5830921

[95] Maharana S, Wang J, Papadopoulos DK, Richter D, Pozniakovsky A, Poser I, et al. RNA buffers the phase separation behavior of prion-like RNA binding proteins. Science. 2018;360(6391):918–921. doi: 10.1126/science.aar7366 29650702 PMC6091854

[96] Wang J, Choi JM, Holehouse AS, Lee HO, Zhang X, Jahnel M, et al. A molecular grammar governing the driving forces for phase separation of prion-like RNA binding proteins. Cell. 2018;174(3):688–699. doi: 10.1016/j.cell.2018.06.006 29961577 PMC6063760

[97] Johnson CN, Sojitra KA, Sohn EJ, Moreno-Romero AK, Baudin A, Xu X, et al. Insights into Molecular Diversity within the FUS/EWS/TAF15 Protein Family: Unraveling Phase Separation of the N-Terminal Low-Complexity Domain from RNA-Binding Protein EWS. Journal of the American Chemical Society. 2024;146(12):8071–8085. doi: 10.1021/jacs.3c12034 38492239 PMC11156192

[98] Babinchak WM, Haider R, Dumm BK, Sarkar P, Surewicz K, Choi JK, et al. The role of liquid–liquid phase separation in aggregation of the TDP-43 low-complexity domain. Journal of Biological Chemistry. 2019;294(16):6306–6317. doi: 10.1074/jbc.RA118.007222 30814253 PMC6484124

[99] Hofweber M, Hutten S, Bourgeois B, Spreitzer E, Niedner-Boblenz A, Schifferer M, et al. Phase separation of FUS is suppressed by its nuclear import receptor and arginine methylation. Cell. 2018;173(3):706–719. doi: 10.1016/j.cell.2018.03.004 29677514

[100] Krainer G, Welsh TJ, Joseph JA, Espinosa JR, Wittmann S, de Csillery E, et al. Reentrant liquid condensate phase of proteins is stabilized by hydrophobic and non-ionic interactions. Nature Communications. 2021;12(1):1–14. doi: 10.1038/s41467-021-21181-9 33597515 PMC7889641

[101] Alberti S, Hyman AA. Biomolecular condensates at the nexus of cellular stress, protein aggregation disease and ageing. Nature reviews Molecular cell biology. 2021;22(3):196–213. doi: 10.1038/s41580-020-00326-6 33510441

[102] Portz B, Lee BL, Shorter J. FUS and TDP-43 phases in health and disease. Trends in biochemical sciences. 2021;46(7):550–563. doi: 10.1016/j.tibs.2020.12.005 33446423 PMC8195841

[103] Jawerth L, Fischer-Friedrich E, Saha S, Wang J, Franzmann T, Zhang X, et al. Protein condensates as aging Maxwell fluids. Science. 2020;370(6522):1317–1323. doi: 10.1126/science.aaw4951 33303613

[104] Chatterjee S, Kan Y, Brzezinski M, Koynov K, Regy RM, Murthy AC, et al. Reversible kinetic trapping of fus biomolecular condensates. Advanced Science. 2022;9(4):2104247. doi: 10.1002/advs.202104247 34862761 PMC8811844

[105] Guenther EL, Cao Q, Trinh H, Lu J, Sawaya MR, Cascio D, et al. Atomic structures of TDP-43 LCD segments and insights into reversible or pathogenic aggregation. Nature structural & molecular biology. 2018;25(6):463–471. doi: 10.1038/s41594-018-0064-2 29786080 PMC5990464

[106] Linsenmeier M, Faltova L, Morelli C, Capasso Palmiero U, Seiffert C, Küffner AM, et al. The interface of condensates of the hnRNPA1 low-complexity domain promotes formation of amyloid fibrils. Nature chemistry. 2023;15(10):1340–1349. doi: 10.1038/s41557-023-01289-9 37749234 PMC10533390

[107] Fisher RS, Elbaum-Garfinkle S. Tunable multiphase dynamics of arginine and lysine liquid condensates. Nature communications. 2020;11(1):1–10. doi: 10.1038/s41467-020-18224-y 32934220 PMC7492283

[108] Sundaravadivelu Devarajan D, Wang J, Szała-Mendyk B, Rekhi S, Nikoubashman A, Kim YC, et al. Sequence-dependent material properties of biomolecular condensates and their relation to dilute phase conformations. Nature Communications. 2024;15(1):1912. doi: 10.1038/s41467-024-46223-w 38429263 PMC10907393

[109] Boeynaems S, Holehouse AS, Weinhardt V, Kovacs D, Van Lindt J, Larabell C, et al. Spontaneous driving forces give rise to protein- RNA condensates with coexisting phases and complex material properties. Proceedings of the National Academy of Sciences. 2019;116(16):7889–7898. doi: 10.1073/pnas.1821038116 30926670 PMC6475405

[110] Ramirez J, Sukumaran SK, Vorselaars B, Likhtman AE. Efficient on the fly calculation of time correlation functions in computer simulations. The Journal of chemical physics. 2010;133(15):154103. doi: 10.1063/1.3491098 20969366

[111] Tejedor AR, Tejedor JR, Ramírez J. Detailed dynamics of discrete Gaussian semiflexible chains with arbitrary stiffness along the contour. The Journal of Chemical Physics. 2022;157(16). doi: 10.1063/5.0112951 36319430

[112] Tejedor AR, Collepardo-Guevara R, Ramirez J, Espinosa JR. Time-Dependent Material Properties of Aging Biomolecular Condensates from Different Viscoelasticity Measurements in Molecular Dynamics Simulations. The Journal of Physical Chemistry B. 2023;127:4441–4459. doi: 10.1021/acs.jpcb.3c01292 37194953 PMC10226127

[113] Deng H, Gao K, Jankovic J. The role of FUS gene variants in neurodegenerative diseases. Nature Reviews Neurology. 2014;10(6):337–348. doi: 10.1038/nrneurol.2014.78 24840975

[114] Wegmann S, Eftekharzadeh B, Tepper K, Zoltowska KM, Bennett RE, Dujardin S, et al. Tau protein liquid–liquid phase separation can initiate tau aggregation. The EMBO journal. 2018;37(7):e98049. doi: 10.15252/embj.201798049 29472250 PMC5881631

[115] Rai SK, Savastano A, Singh P, Mukhopadhyay S, Zweckstetter M. Liquid–liquid phase separation of tau: From molecular biophysics to physiology and disease. Protein Science. 2021;30(7):1294–1314. doi: 10.1002/pro.4093 33930220 PMC8197432

[116] Galvanetto N, Ivanović MT, Chowdhury A, Sottini A, Nüesch MF, Nettels D, et al. Extreme dynamics in a biomolecular condensate. Nature. 2023;619(7971):876–883. doi: 10.1038/s41586-023-06329-5 37468629 PMC11508043

[117] Choi JM, Pappu RV. The Stickers and Spacers Framework for Describing Phase Behavior of Multivalent Intrinsically Disordered Proteins. Biophysical Journal. 2020;118(3):492a. doi: 10.1016/j.bpj.2019.11.272331839263

[118] Choi UB, Sanabria H, Smirnova T, Bowen ME, Weninger KR. Spontaneous switching among conformational ensembles in intrinsically disordered proteins. Biomolecules. 2019;9(3):114. doi: 10.3390/biom9030114 30909517 PMC6468417

[119] Zhou HX, Kota D, Qin S, Prasad R. Fundamental Aspects of Phase-Separated Biomolecular Condensates. Chemical Reviews. 2024;. doi: 10.1021/acs.chemrev.4c00138 38885177 PMC11260227

[120] Regy RM, Dignon GL, Zheng W, Kim YC, Mittal J. Sequence dependent phase separation of protein-polynucleotide mixtures elucidated using molecular simulations. Nucleic acids research. 2020;48(22):12593–12603. doi: 10.1093/nar/gkaa1099 33264400 PMC7736803

[121] Ashbaugh HS, Hatch HW. Natively unfolded protein stability as a coil-to-globule transition in charge/hydropathy space. Journal of the American Chemical Society. 2008;130(29):9536–9542. doi: 10.1021/ja802124e 18576630

[122] Lorentz HA. On the application of the virial theorem in the kinetic theory of gases. Annals of physics. 1881;248(1):127–136. doi: 10.1002/andp.18812480110

[123] Berthelot D. Sur le mélange des gaz. Compt Rendus. 1898;126(3):15.

[124] Plimpton S. Fast parallel algorithms for short-range molecular dynamics. Journal of computational physics. 1995;117(1):1–19. doi: 10.1006/jcph.1995.1039

[125] Thompson AP, Aktulga HM, Berger R, Bolintineanu DS, Brown WM, Crozier PS, et al. LAMMPS—a flexible simulation tool for particle-based materials modeling at the atomic, meso, and continuum scales. Comp Phys Comm. 2022;271:108171. doi: 10.1016/j.cpc.2021.108171

[126] Schneider T, Stoll E. Molecular-dynamics study of a three-dimensional one-component model for distortive phase transitions. Physical Review B. 1978;17(3):1302. doi: 10.1103/PhysRevB.17.1302

[127] García Fernández R, Abascal JL, Vega C. The melting point of ice Ih for common water models calculated from direct coexistence of the solid-liquid interface. The Journal of chemical physics. 2006;124(14). 10.1063/1.2183308 16626213

[128] Blas FJ, MacDowell LG, de Miguel E, Jackson G. Vapor-liquid interfacial properties of fully flexible Lennard-Jones chains. The Journal of chemical physics. 2008;129(14). doi: 10.1063/1.2989115 19045161

[129] Espinosa JR, Sanz E, Valeriani C, Vega C. On fluid-solid direct coexistence simulations: The pseudo-hard sphere model. The Journal of chemical physics. 2013;139(14). doi: 10.1063/1.4823499 24116630

[130] Rowlinson JS, Widom B. Molecular theory of capillarity. Courier Corporation; 2013.

[131] Rubinstein M, Colby RH. Polymer physics. Oxford university press; 2003.

[132] Likhtman AE, Sukumaran SK, Ramirez J. Linear viscoelasticity from molecular dynamics simulation of entangled polymers. Macromolecules. 2007;40(18):6748–6757. doi: 10.1021/ma070843b

[133] Tejedor AR, Tejedor JR, Ramírez J. Detailed dynamics of discrete Gaussian semiflexible chains with arbitrary stiffness along the contour. The Journal of Chemical Physics. 2022;157(16). doi: 10.1063/5.0112951 36319430

[134] Bagheriasl D, Carreau PJ, Riedl B, Dubois C, Hamad WY. Shear rheology of polylactide (PLA)–cellulose nanocrystal (CNC) nanocomposites. Cellulose. 2016;23:1885–1897. doi: 10.1007/s10570-016-0914-1

[135] Fisher RS, Elbaum-Garfinkle S. Tau biomolecular condensates exhibit length scale dependent aging. Biophysical Journal. 2022;121(3):147a. doi: 10.1016/j.bpj.2021.11.1992

